# Supplementary material for: AI-guided additive scoring model for differential diagnosis of primary liver cancer
Source: JHEP Rep. 2026 Mar 25;8(6):101826. doi: 10.1016/j.jhepr.2026.101826 (PMC13158401; doi:10.1016/j.jhepr.2026.101826)
Supplement: Multimedia component 1 [file mmc1.pdf]

AI-guided additive scoring model for differential diagnosis of primary liver cancer

Rebekka J.S. Salzmann, Tudor Mocan, Arnulf G. Willms, Angelina Klein, Robert Schwab, Emil Mois, Cristiana Grapa, Lavinia Patricia Mocan, Rares Craciun, Zeno Sparchez, Ingo G.H. Schmidt-Wolf, Jan Best, Hartmut H. Schmidt, Marcin Krawczyk[7], Simon C. Robson, Veronika Lukacs-Kornek, Miroslaw T. Kornek

Table of contents

Supplementary figures……………………………………………………………………2

Supplementary tables………………………………………………………………..………3

Supplementary figures

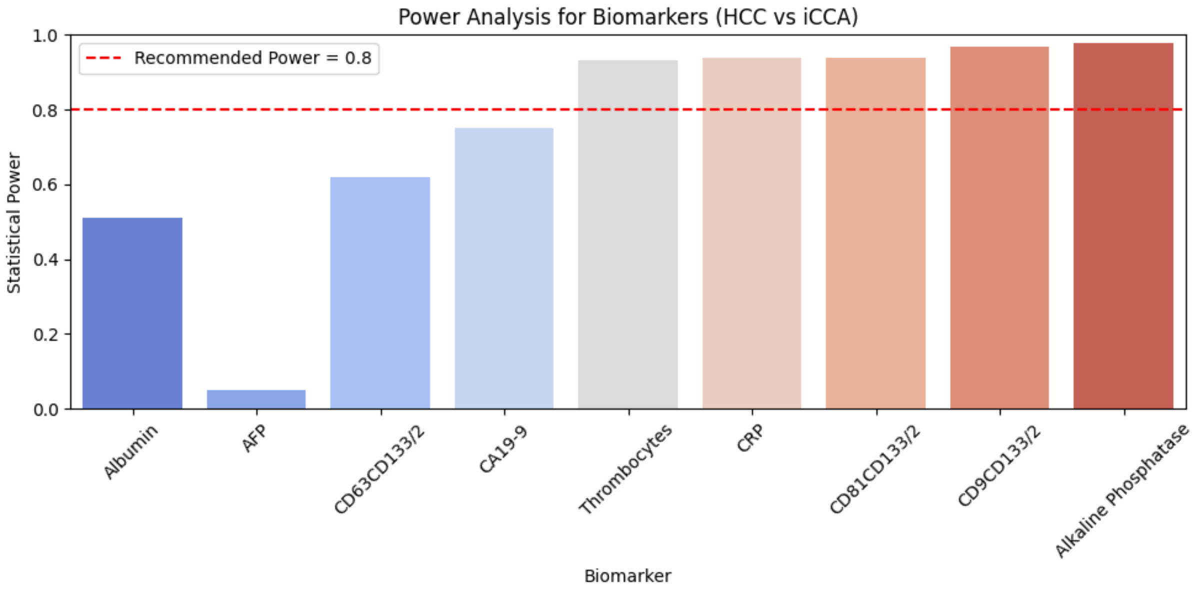

Fig. S1 Statistical power analysis for individual biomarkers (HCC vs iCCA). *The post-hoc power estimation was performed for each biomarker comparison using effect sizes derived from Mann–Whitney U tests (n = 25 per group). Bars indicate the achieved statistical power for detecting group differences between hepatocellular carcinoma (HCC) and intrahepatic cholangiocarcinoma (iCCA). The red dashed line marks the recommended threshold for adequate power (0.8). Most parameters—particularly Alkaline Phosphatase, CD9⁺CD133/2⁺, CD81⁺CD133/2⁺, CRP, and Thrombocytes—exceeded this threshold, indicating sufficient sensitivity to detect true differences. AFP and Albumin showed lower power due to high intra-group variability and limited effect size.*

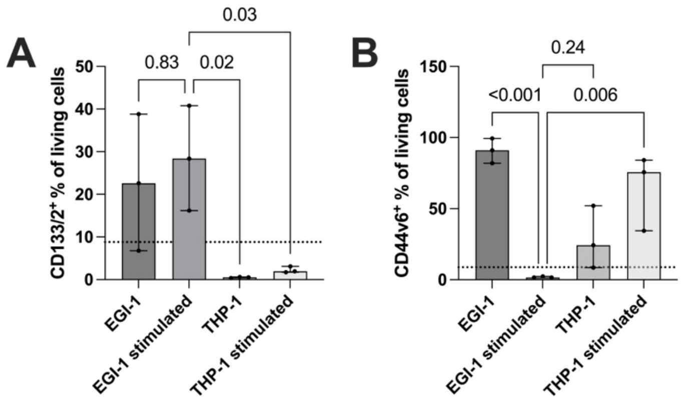

Fig. S2 Validation of CD133/2 and CD44v6 antibody candidates on EGI-1 cholangiocarcinoma cells and THP-1 monocytic cells. *Biological triplicates of unstimulated and stimulated EGI-1 cells and unstimulated and stimulated THP-1 cells were immunolabeled with candidate antibodies against CD133/2 (PE) and CD44v6 (APC) and analyzed by flow cytometry for surface expression. (A) CD133/2 surface expression on EGI-1 cells under standard culture conditions and after starvation (FBS deprivation), and on THP-1 cells under standard conditions and after cytokine stimulation. Starvation of EGI-1 cells resulted in a significant increase in CD133/2 expression compared to unstimulated EGI-1 cells ($p = 0.03$). No significant CD133/2 expression was detected on THP-1 cells under either condition ($p = 0.83$ and $p = 0.02$, respectively). (B) CD44v6 surface expression on EGI-1 and THP-1 cells. EGI-1 cells showed significantly higher CD44v6 expression compared to THP-1 cells under standard culture conditions ($p < 0.001$). Cytokine stimulation of THP-1 cells with IL-4 and IL-13 for 72 h resulted in a significant induction of CD44v6 expression compared to unstimulated THP-1 cells ($p = 0.006$), while no significant change was observed in EGI-1 cells following starvation ($p = 0.24$). Bars represent median values with 95% confidence intervals. Statistical analysis was performed using GraphPad Prism (version 10.4.1, GraphPad Software, USA). Group comparisons were conducted using one-way ANOVA assuming Gaussian distribution, followed by Dunnett's multiple comparisons post-test. All tests were two-sided with a confidence level of 95%.*

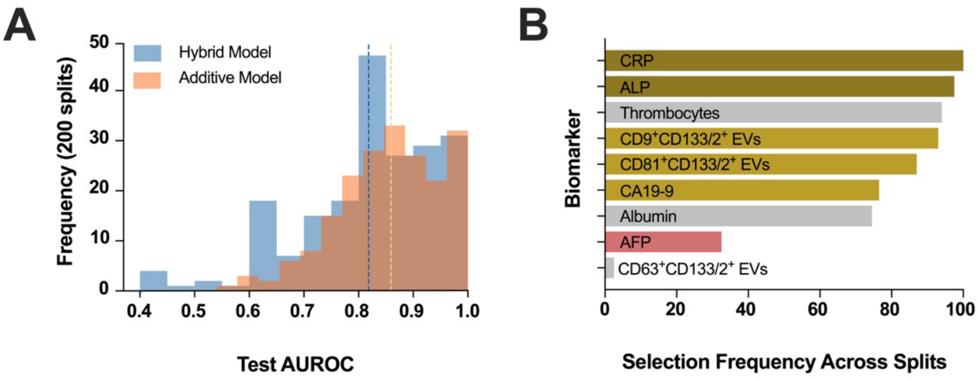

Fig. S3 Stability of diagnostic performance and feature selection across 200 repeated random train–test splits. *(A) Distribution of test AUROC values across 200 repeated random 80:20 train–test splits for the hybrid logistic regression model (continuous predictors) and the translated additive scoring model (binarized ROC–Youden thresholds). Dashed vertical lines indicate the mean test AUROC across splits for each model (hybrid mean = 0.818; additive mean = 0.859). (B) LASSO feature-selection frequency across the same 200 random splits, shown as the percentage of splits in which each biomarker was selected during model training. Core markers (CRP, alkaline phosphatase, thrombocytes, CD9^{+}CD133/2^{+} EVs, CD81^{+}CD133/2^{+} EVs) demonstrated consistently high selection frequencies, whereas AFP and CD63^{+}CD133/2^{+} EVs were selected less frequently.*

Supplementary tables

Table S1 Patient demographics and baseline clinical characteristics of the iCCA and HCC cohorts. *Continuous variables are presented as median [interquartile range, IQR], and categorical variables as n (%). P-values for continuous variables were calculated using the two-sided Mann–Whitney U test; p-values for categorical variables were calculated using Fisher's exact test. Exact p-values are reported in the table. Blank cells indicate insufficient data for statistical comparison. Barcelona Clinic Liver Cancer (BCLC) classification applies only to HCC patients, while American Joint Committee on Cancer (AJCC) staging applies only to iCCA patients.*

Variable	iCCA (n=25)	HCC (n=25)	p-value
Age	64.00 [58.25–68.00]	66.00 [61.25–70.75]	0.42
CRP	2.83 [0.88–7.83]	0.59 [0.43–0.77]	0.001
ALP	690.00 [456.00–1377.00]	271.00 [212.00–380.00]	<0.001
Albumin	3.50 [3.20–3.80]	3.70 [3.41–4.00]	0.05
Thrombocytes	231.00 [187.00–279.00]	135.00 [90.00–178.00]	<0.001
AFP	3.90 [2.70–21.00]	16.70 [6.80–57.70]	0.93
CA19-9	150.80 [19.70–200.00]	11.40 [5.70–56.70]	0.005
Gender: male	17 (68.0%)	16 (64.0%)	1.00
Gender: female	8 (32.0%)	9 (36.0%)	1.00
BCLC A		52.0%	
BCLC B		B: 20.0%	
BCLC C		C: 16.0%	
BCLC D		D: 12.0%	
AJCC stages	IB: 12.0%; II: 8.0%; IIIA: 4.0%; IIIB: 16.0%; IV: 36.0%; IVA: 8.0%; IVB: 16.0%		

Table S2 List of antibodies and isotypes used for cell- surface flow cytometric analysis and SP-IRIS*. The antibodies were titrated against their matching isotype controls before use and applied according to the manufacturers' instructions.*

Antibody (anti-human)	Conjugate	Clone	Catalog#	Vendor	Concentration [µg/µL]
CD133/2	PE	REA820	130-112-195	Miltenyi Biotec	0.15
Isotype	PE	REA293	130-118-347	Miltenyi Biotec	0.15
CD44v6	APC	REA706	130-111-238	Miltenyi Biotec	0.1
Isotype	APC	REA293	130-113-446	Miltenyi Biotec	0.02

Table S3. Data completeness across biomarkers and cohorts: *Overview of data completeness for all biomarkers included in the analysis, stratified by disease cohort (HCC and iCCA). Expected indicates the theoretical maximum number of measurements per cohort (n = 25 each), while Available reflects the number of measurements present in the final analysis dataset. Missing values were limited and primarily affected routine serological parameters, whereas all extracellular vesicle–derived markers were fully available across both cohorts.*

Biomarker	iCCA Expected (n=25)	iCCA Available (n)	HCC Expected (n=25)	HCC Available (n)	Missing total (n)
AFP	25	25	25	23	2
Albumin	25	25	25	25	0
ALP	25	25	25	25	0
CA19-9	25	22	25	22	6
CRP	25	25	25	25	0
Thrombocytes	25	25	25	25	0
$CD9^+CD133/2^+$ EVs	25	25	25	25	0
$CD63^+CD133/2^+$ EVs	25	25	25	25	0
$CD81^+CD133/2^+$ EVs	25	25	25	25	0

Table S4 Supplementary Table 4. Diagnostic performance of hybrid logistic regression models combining extracellular vesicle–derived and routine serological biomarkers for differentiation of HCC and iCCA. *AUROC values were computed in Python (scikit-learn) from receiver operating characteristic (ROC) curves of the respective model scores. Sensitivity and specificity were derived from the confusion matrix at the ROC-derived Youden-index threshold. P-values were calculated using a two-sided Pearson chi-square test applied to the 2×2 contingency table of predicted class versus histopathological ground truth (TP/FN/FP/TN), testing for independence between model prediction and disease entity.*

Model	AUROC	Sensitivity	Specificity	Chi-square p-value
With AFP (LASSO/PCA)	0.857	0.714	1.000	0.050
Without AFP (LASSO/PCA)	0.905	0.857	1.000	0.050

Table S5 Performance metrics of the additive scoring system derived from the LASSO/PCA hybrid models for differentiation of iCCA from HCC in LR-M lesions. *AUROC values were computed in Python (scikit-learn) from ROC curves of the respective total additive scores. The optimal total score cutoff for each model was determined using Youden's index (maximum of TPR – FPR). Sensitivity and specificity were calculated from the resulting confusion matrix at the selected cutoff. P-values were obtained using a two-sided Pearson chi-square test applied to the 2×2 contingency table of predicted class versus histopathological ground truth (TP/FN/FP/TN), testing for independence between model-based classification and disease entity.*

Model	AUROC	Sensitivity	Specificity	Chi-square p-value
With AFP (LASSO/PCA Final Model)	0.962	0.76	1.000	<0.0001
Without AFP (LASSO/PCA Final Model)	0.952	0.76	1.000	<0.0001

Table S6 Monte Carlo robustness analysis of the PRISM-based 5-point additive scoring system. *To assess the stability of the PRISM-derived score under measurement variability, marker values were perturbed by multiplicative uniform noise at ±5%, ±10%, and ±20% (independently per marker and per sample). For each noise level, 500 Monte Carlo iterations were performed. In each iteration, the total additive score was recalculated using the fixed PRISM cut-offs, and AUROC was recomputed from*

the resulting total scores. The table reports mean AUROC, standard deviation, and minimum/maximum AUROC across iterations. No hypothesis testing was performed; therefore, no p-values apply.

Noise Level	Mean AUROC	SD (AUROC)	Min AUROC	Max AUROC
±5 %	0.894	0.008	0.877	0.914
±10 %	0.894	0.010	0.87	0.925
±20 %	0.891	0.015	0.846	0.933

Table S7. Bootstrap-derived uncertainty of cohort-derived ROC–Youden cut-offs (2,000 iterations). *Cut-off stability was evaluated using stratified bootstrap resampling (2,000 iterations). For each biomarker, the table reports the median bootstrap-derived cut-off, the 2.5% and 97.5% percentile interval, and the percentage of bootstrap iterations in which the direction of effect (higher values associated with iCCA) remained consistent. Stability classification reflects combined assessment of directional consistency and relative dispersion of the bootstrap cut-off distribution. AFP was included for completeness but is fixed at 20 ng/mL in guideline-based analyses.*

Biomarker	Bootstrap Median Cut-off	2.5% Percentile	97.5% Percentile	Direction Consistency (%)	Bootstrap Stability
AFP	6.7	2.8	32.0	7.7	unstable
Albumin	3.5	3.0	4.0	2.6	unstable
ALP	456.0	456.0	912.0	100.0	stable
CA19-9	117.9	3.6	171.1	95.0	unstable
Thrombocytes	167.0	166.0	217.0	100.0	stable
$CD9^+CD133/2^+$ EVs	5292000.0	2168633.3	11687000.0	100.0	stable
$CD81^+CD133/2^+$ EVs	1582000.0	161000.0	3700000.0	100.0	moderate

Table S8 Cox proportional hazards models and assessment of proportional hazards assumptions for CD133/2-positive small extracellular vesicle subpopulations in LR-M patients. *Univariable Cox proportional hazards models were performed separately within HCC and iCCA subgroups (n = 25 each) to evaluate the association between above- versus ≤-median small extracellular vesicle (EV) levels and overall survival. Hazard ratios (HR) with 95% confidence intervals (CI) are shown. The proportional hazards (PH) assumption was formally tested using Schoenfeld residual–based tests (rank transformation). Corresponding p-values are reported. A p-value > 0.05 indicates no evidence of violation of the PH assumption. No significant deviations from proportional hazards were detected in any model.*

Model	n	Events	HR	95% CI	Schoenfeld p	PH violated?
HCC $CD9^+CD133/2^+$	25	20	1.89	0.76–4.70	0.444	No
HCC $CD81^+CD133/2^+$	25	20	2.88	1.11–7.43	0.893	No
iCCA $CD9^+CD133/2^+$	25	23	3.89	1.43–10.58	0.586	No
iCCA $CD81^+CD133/2^+$	25	23	2.16	0.87–5.34	0.337	No
