# Supplementary material for: AI-guided additive scoring model for differential diagnosis of primary liver cancer
Source: JHEP Rep. 2026 Mar 25;8(6):101826. doi: 10.1016/j.jhepr.2026.101826 (PMC13158401; doi:10.1016/j.jhepr.2026.101826)
Supplement: Multimedia component 2 [file mmc2.docx]

**JHEP Reports**

**CTAT methods**

Tables for a “Complete, Transparent, Accurate and Timely account” (CTAT) are now mandatory for all revised submissions. The aim is to enhance the reproducibility of methods.

- Only include the parts relevant to your study
- Refer to the CTAT in the main text as ‘Supplementary CTAT Table’
- Do not add subheadings
- Add as many rows as needed to include all information
- Only include one item per row

**If the CTAT form is not relevant to your study, please outline the reasons why:**

|  |
| --- |

- 1. **Antibodies**

| **Name** | **Citation** | **Supplier** | **Cat no.** | **Clone no.** |
| --- | --- | --- | --- | --- |
| **CD133/2 PE** | **https://www.miltenyibiotec.com/DE-en/products/cd133-2-antibody-anti-human-reafinity-rea820.html#conjugate=pe:size=100-tests-in-200-ul** | **Miltenyi Biotec** | **130-112-195** | **REA820** |
| **REA Isotype PE** | **https://www.miltenyibiotec.com/DE-en/products/rea-control-antibody-i-human-igg1-reafinity-rea293.html#conjugate=pe:size=100-tests-in-200-ul** | **Miltenyi Biotec** | **130-118-347** | **REA293** |
| **CD44v6 APC** | **https://www.miltenyibiotec.com/DE-en/products/cd44v6-antibody-anti-human-reafinity-rea706.html#Conjugate=APC:size=100-tests-in-200-uL** | **Miltenyi Biotec** | **130-111-238** | **REA706** |
| **REA Isorype APC** | **https://www.miltenyibiotec.com/DE-en/products/rea-control-antibody-human-igg1-reafinity-rea293.html#Conjugate=APC:size=30-ug-in-200-uL** | **Miltenyi Biotec** | **130-113-446** | **REA293** |

- 1. **Cell lines**

| **Name** | **Citation** | **Supplier** | **Cat no.** | **Passage no.** | **Authentication test method** |
| --- | --- | --- | --- | --- | --- |
| **THP-1** | **https://www.hoelzel-biotech.com/de/cells-and-cell-lines-cls-300356-thp-1.html** | **CLS, Germany** | **#300356** | **23** | **validated by the supplier. Mail to: service@clsgmbh.de** |
| **EGI-1** | **https://www.dsmz.de/collection/catalogue/details/culture/ACC-385** | **DSMZ; Germany** | **#ACC385** | **16** | **STR analysis according to the global standard ANSI/ATCC ASN-0002.1-2021 (2021) resulted in an authentic STR profile of the reference STR database** |

- 1. **Organisms**

| **Name** | **Citation** | **Supplier** | **Strain** | **Sex** | **Age** | **Overall n number** |
| --- | --- | --- | --- | --- | --- | --- |
|  |  |  |  |  |  |  |

- 1. **Sequence based reagents**

| **Name** | **Sequence** | **Supplier** |
| --- | --- | --- |
|  |  |  |

- 1. **Biological samples**

| **Description** | **Source** | **Identifier** |
| --- | --- | --- |
| **Details see MATERIAL AND METHODS - Human study cohort -** | **Details see MATERIAL AND METHODS - Human study cohort -** | **Details see MATERIAL AND METHODS - Human study cohort -** |

- 1. **Deposited data**

| **Name of repository** | **Identifier** | **Link** |
| --- | --- | --- |
|  |  |  |

- 1. **Software**

| **Software name** | **Manufacturer** | **Version** |
| --- | --- | --- |
| **GraphPad Prism -** | **GraphPad Software, La Jolla, USA** | **v10.4.1** |
| **Google Colab** | **Google LLC, 1600 Amphitheatre Parkway, Mountain View, California 94043** | **CPython 3.11.5, pandas 2.2, numpy 1.26.4, scikit-learn 1.6.1, matplotlib 3.10.0, seaborn 0.13.2, and scipy 1.14.1 and lifelines 0.30.0** |

- 1. **Other (*e.g*. drugs, proteins, vectors etc.)**

|  |  |  |
| --- | --- | --- |
|  |  |  |

- 1. **Please provide the details of the corresponding methods author for the manuscript:**

| **Dr. Miroslaw T. Kornek** is the corresponding methods author. He led the AI-assisted re-analysis, curated the dataset, and collaborated with OpenAI on Python-based modelling and validation workflows. He is responsible for the integrity and reproducibility of the analytical workflow and data analysis. Rebekka J.S. Salzmann is responsible for all small EV measurements and cell culture work and flow cytometric analysis. |
| --- |

**2.0 Please confirm for randomised controlled trials all versions of the clinical protocol are included in the submission. These will be published online as supplementary information.**

|  |
| --- |
