# Supplementary material for: AI-guided additive scoring model for differential diagnosis of primary liver cancer
Source: JHEP Rep. 2026 Mar 25;8(6):101826. doi: 10.1016/j.jhepr.2026.101826 (PMC13158401; doi:10.1016/j.jhepr.2026.101826)
Supplement: Multimedia component 4 [file mmc4.pdf]

AI-guided additive scoring model for differential diagnosis of primary liver cancer

Authors

Rebekka J.S. Salzmann, Tudor Mocan, Arnulf G. Willms, …, Simon C. Robson, Veronika Lukacs-Kornek, Miroslaw T. Kornek

Correspondence

vlukacsk@uni-bonn.de (V. Lukacs-Kornek), mirokor@uni-bonn.de (M.T. Kornek).

Graphical abstract

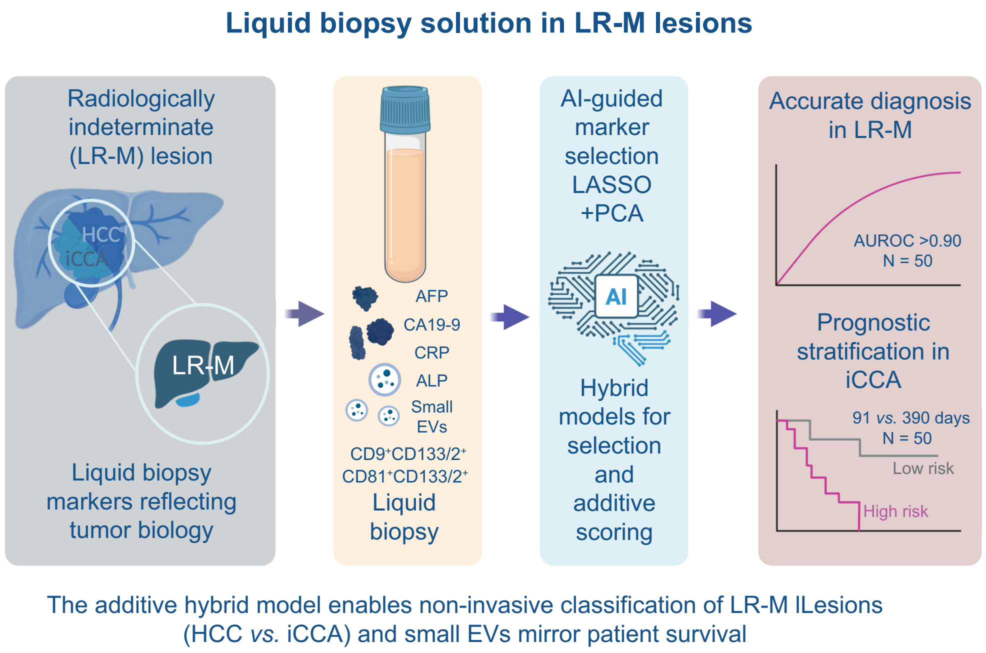

Highlights:

- A hybrid model translated into a simple additive score for LR-M differentiation.
- Small EV phenotyping was combined with serum markers for minimally invasive classification.
- Model showed stable discrimination across bootstrapping and Monte Carlo perturbation (AUROC ~0.91).
- Elevated CD9⁺CD133/2⁺ EVs linked to shorter survival in iCCA.
- ML-guided hybrid model converted into an interpretable additive LR-M score.

Impact and Implications:

This study provides a scientific rationale for integrating small EV phenotyping with conventional serum biomarkers to improve minimally invasive differentiation between HCC and iCCA in LR-M lesions. By translating a machine learning (ML)-derived hybrid model into a simple additive scoring system, we demonstrate how ML can yield clinically interpretable tools that bridge computational discovery and bedside application. The findings are particularly relevant for hepatologists, oncologists, and radiologists managing patients in whom imaging remains indeterminate and biopsy carries procedural risk. The 5-point model can be applied using routine laboratory values, providing a paper-and-pencil diagnostic aid that requires no specialized software or hardware. Although external multicenter validation is necessary given the cohort size, this approach illustrates how transparent, interpretable ML can support precision diagnostics in primary liver cancer.

https://doi.org/10.1016/j.jhepr.2026.101826
© 2026 The Author(s). Published by Elsevier B.V. on behalf of European Association for the Study of the Liver (EASL). This is an open access article under the CC BY license (http://creativecommons.org/licenses/by/4.0/).

AI-guided additive scoring model for differential diagnosis of primary liver cancer

Rebekka J.S. Salzmann[1,2,†], **Tudor Mocan**[3,4,†], **Arnulf G. Willms**[5,†], Angelina Klein[5], Robert Schwab[5], Emil Mois[3], Cristiana Grapa[3], Lavinia Patricia Mocan[3], Rares Craciun[3], Zeno Sparchez[3], Ingo G.H. Schmidt-Wolf[6], Jan Best[7], Hartmut H. Schmidt[7], Marcin Krawczyk[7,8], Simon C. Robson[9], Veronika Lukacs-Kornek[2,*], Miroslaw T. Kornek[2,5,*]

JHEP Reports **2026**. vol. 8 | 1–15

Check for updates

Background & Aims: Within the Liver Imaging Reporting and Data System (LI-RADS), LI-RADS Malignant (LR-M) lesions remain diagnostically challenging: imaging indicates malignancy, but often fails to distinguish hepatocellular carcinoma (HCC) from intrahepatic cholangiocarcinoma (iCCA). Established serum-based tools, such as GALAD, are optimized for HCC, but are not designed to resolve entity ambiguity within LR-M. In this pilot proof of concept study, we investigated whether small extracellular vesicles (EVs) integrated with routine serological markers could resolve LR-M lesion situation.

Methods: A rare LR-M cohort (HCC = 25, iCCA = 25) was evaluated using least absolute shrinkage and selection operator (LASSO) logistic regression and PCA to prioritize informative biomarkers. Hybrid models combined $CD9^{+}CD133/2^{+}$ and $CD81^{+}CD133/2^{+}$ EVs with alkaline phosphatase, serum C-reactive protein, CA19-9, and optional alpha-fetoprotein (AFP). Models were internally validated using an 80:20 train–test split and assessed with bootstrap and Monte Carlo perturbation (±5–20%).

Results: Individual markers demonstrated limited discrimination (AUROC ≤0.82). Hybrid logistic regression models showed moderate internal discrimination (AUROC 0.86 with AFP; 0.91 without AFP). Translation into additive scoring systems using ROC–Youden-derived cut-offs yielded high internal AUROC estimates (*e.g.* 0.95–0.96), although these remain internally validated. A simplified 5-point PRISM score retained comparable discriminatory performance (AUROC ~0.91). In an exploratory survival analysis among patients with iCCA (n = 25), those with above-median $CD9^{+}CD133/2^{+}$ EV levels (n = 13) had shorter overall survival (median 91 *vs.* 389 days; HR: 2.80, 95% CI: 1.16–6.74; $p = 0.005$).

Conclusions: Integrated EV and serological profiling could enable minimally invasive differentiation between HCC and iCCA within LR-M lesions. By transforming machine learning (ML)-guided model discovery into a clinically interpretable paper-and-pencil additive score, we illustrate a translational pathway from computational discovery to practical application.

© 2026 The Author(s). Published by Elsevier B.V. on behalf of European Association for the Study of the Liver (EASL). This is an open access article under the CC BY license (http://creativecommons.org/licenses/by/4.0/).

Introduction

Primary liver cancer ranks as the fifth most prevalent cancer globally, is increasing in prevalence, and is already the second leading cause of cancer-related mortality.[1,2] The 5-year survival rate for liver cancer is only 20%, in stark contrast to the average survival rate of 68% across all cancer types, as reported in 2026.[3] Many hepatic cancer cases are diagnosed at an advanced stage because of late clinical presentation, rendering surgical resection, the sole curative treatment option, unavailable.[4–6] Given the potentially severe implications of liver cancer, the development and application of minimally invasive, rapid, and reproducible biomarkers for early diagnosis, prognosis, and screening, supplementing imaging and/or making conventional biopsies obsolete, are vital. Robust diagnosis and reliable prognosis have the potential to enhance treatment outcomes and significantly improve survival rates for individuals with liver cancer.[7,8]

Primary liver cancer is a distinctive subtype of liver cancer that develops from the hepatic cells themselves.[9,10] The liver primarily comprises hepatocytes, but contains various other cell types, such as endothelial cells forming blood vessels, and epithelial cells that line the bile ducts. There are various types of primary liver cancer, with hepatocellular carcinoma (HCC) being the most prevalent, followed by intrahepatic cholangiocarcinoma (iCCA).[11,12] Differentiating between HCC and iCCA is important for tailoring therapeutic options, but can be particularly challenging, even with the use of minimally invasive imaging techniques, such as computed tomography (CT) and magnetic resonance imaging (MRI) with the use of the Liver Imaging Reporting and Data System (LI-RADS) classification. In instances where patients fall into the LI-RADS category M (*i.*

* Corresponding authors. Addresses: Institute of Molecular Medicine and Experimental Immunology, University Bonn, Venusberg Campus 1, Geb. 12, BMZII, 53127 Bonn, Germany (V. Lukacs-Kornek). German Armed Forces Hospital Koblenz, Department of General, Visceral and Thoracic Surgery, Rübenacher Str. 170, 56072 Koblenz, Germany (M.T. Kornek).
E-mail addresses: vlukacsk@uni-bonn.de (V. Lukacs-Kornek), mirokor@uni-bonn.de (M.T. Kornek).
† These authors contributed equally.
https://doi.org/10.1016/j.jhepr.2026.101826

e. LR-M, indicative of definitive or probable malignancy, not specific for HCC), a potentially hazardous liver biopsy is recommended to confirm the diagnosis[13] if robust minimally invasive biomarkers are absent.

In this study, we investigated the diagnostic benefit of combining routinely available serological parameters with extracellular vesicle (EV) phenotyping and quantification. Rather than aiming for broad cancer detection or pan-organ classification, we address a focused and clinically relevant problem within a well-defined liver cancer cohort: the differentiation between HCC and iCCA in patients with LI-RADS LR-M lesions, where malignancy is established but tumor entity remains uncertain. In such LR-M cases, representing ~10% of liver cancer cases, the well-established standard operating procedures (SOPs) and staging systems relying on morphology and location are not conclusive, based on minimally invasive visual assessment by CT and MRI.[13] Only an invasive biopsy will result in a clear differentiation and diagnosis. We hypothesized that blood-liquid biopsy markers, such as small EVs and routinely available serological markers, might have the potential to act as liquid biopsies to differentiate between HCC and iCCA in LR-M cases.

EVs have been known for decades and were initially described as ‘platelet dust’ in early electron microscopy studies.[14] Their relevance for liquid biopsy emerged during the early 2000s, when tumor-derived vesicles carrying proteins and nucleic acids were detected in the circulation.[15] Since then, EVs have been extensively explored as biomarkers across a broad range of diseases, including cancer, neurological disorders, and infectious conditions.[16] They are lipid bilayer-enclosed particles decorated with membrane-associated proteins, such as tetraspanins, integrins, and proteoglycans, the composition of which reflects the physiological or pathological state of the parent cell.[17–19] They are released by many cell types, including immune cells, stem cells, and cancer cells, and participate in intercellular communication and disease progression.[20–26] Based on their biogenesis and size, EVs are broadly classified into small EVs (commonly referred to as exosomes; ~30–150 nm), which originate from the endosomal system, and large EVs (microvesicles/ectosomes), which are generated by direct outward budding of the plasma membrane.[27,28]

Previously, we reported synergistic effects in separating samples from HCC from CCA (extrahepatic and intrahepatic) taking advantage of AnnV$^+$CD133$^+$gp38$^+$large EVs and alpha-fetoprotein (AFP).[29] Here, we investigated the performance of CD133/2$^+$ small EVs and their subpopulations (CD9$^+$CD133/2$^+$, CD63$^+$CD133/2$^+$, and CD81$^+$CD133/2$^+$) in differentiating HCC from iCCA in patients classified as LR-M under the LI-RADS.

Material/patients and methods

Ethics: patient samples

This study received approval from the Ethics Committee of the Human Serum Collecting Institution in Cluj-Napoca, Romania (3042/07.03.2018). It adhered to the revised Declaration of Helsinki as well as the 2018 Declaration of Istanbul regarding research involving human volunteers. The research objectives complied with international regulations as well as primary and secondary legislation. Participation in the study was contingent upon the signing of informed written consent. Patient data were handled in accordance with the European Union’s General Data Protection Regulation. All patients provided their informed consent.

Human study cohort

Table S1 summarizes the baseline characteristics of the patients with HCC and iCCA in the LR-M category. A key distinction between the two cohorts is that iCCA is typically diagnosed at a more advanced stage of disease progression and HCC is cofounded with liver cirrhosis, as reflected in our patient collective and assessed by cancer-specific scoring systems, that is, Barcelona Clinic Liver Cancer (BCLC) staging for HCC and the American Joint Committee on Cancer (AJCC) staging for iCCA. The final diagnosis for each case was established through imaging techniques, followed by histopathological confirmation of the liver malignancy. Median age was comparable between iCCA and HCC (64.0 [58.25–68.00] *vs.* 66.0 [61.25–70.75] years, p = 0.4174). Inflammatory and cholestatic parameters were significantly elevated in iCCA, including serum C-reactive protein (CRP; 2.83 *vs.* 0.59 mg/L, p = 0.0004) and alkaline phosphatase (ALP; 690 *vs.* 271 U/L, p = 0.0002). Thrombocyte counts were also higher in iCCA (231 *vs.* 135 × 10^3/µl, p = 0.0011), whereas AFP was increased in HCC (16.7 *vs.* 3.9 ng/ml, p = 0.0456) and CA19-9 in iCCA (150.8 *vs.* 11.4 U/ml, p = 0.0275). Gender distribution was balanced, and disease staging reflected typical patterns, with early-stage dominance in HCC (BCLC A: 52%) and advanced stages in iCCA (AJCC IV: 36%).

Blood processing

Blood samples were collected in tubes containing serum separator gel (BD Vacutainer, Franklin Lakes, NJ, USA) and processed in accordance with the manufacturer’s instructions. Serum aliquots were promptly stored at -80 °C until subsequent analysis. Shipment of serum samples was performed on dry ice to avoid thawing of samples. The samples were transferred to -80 °C upon arrival and remained frozen until small EVs were isolated from the serum.

Human cell lines

The cell lines THP-1 (CLS, Baden Württemberg, Germany, #300356) and EGI-1 (DSMZ, Braunschweig, Germany, #ACC385) were cultured in tissue culture flasks T-75 or T-175 (Sarstedt, Rheinbach, Germany) in growth medium comprising RPMI-1640 GlutaMAX (Thermo Fisher Scientific, Gibco, Waltham, USA) supplemented with 10% (v/v) heat inactivated FBS (Thermo Fisher Scientific, Gibco) and 1% (v/v) penicillin-streptomycin (10,000 U/ml, Thermo Fisher Scientific, Gibco) at 37 °C and 5% CO_2 in a humidified incubator.

THP-1 stimulation with IL-4 and IL-13

THP-1 cells were seeded at a density of 0.8 × 10^6 cells/ml and incubated for 72 h in growth medium supplemented with 50 ng/ml PMA (100 µg/ml, Merck, Sigma-Aldrich, Darmstadt, Germany). Subsequently the medium was removed, and cells

were gently washed with PBS, pH 7.4 (Thermo Fisher Scientific, Gibco). The cells were allowed to rest in fresh growth medium for 48 h. The growth medium was renewed and supplemented with 50 ng/ml human IL-4, *Escherichia coli* derived (100 μg/ml, Bio-Techne®, R&D systems, Minneapolis, MN, USA) and 50 ng/ml human IL-13, *E. coli* derived (100 μg/ml, Bio-Techne®, R&D systems) and cells were further incubated for 72 h. Subsequently, the cell culture supernatant was collected for EV isolation and the cells were harvested and subjected to flow cytometric analysis.

EGI-1 stimulation by nutrient deprivation

EGI-1 cells were seeded at a density of 6 × 10^4 cells/cm^2 and incubated for 24 h in RPMI-1640 GlutaMAX (Thermo Fisher Scientific, Gibco) supplemented with 1% (v/v) P/S (10,000 U/ml, Thermo Fisher Scientific, Gibco) at 37 °C and 5% CO_2 in a humidified incubator. Subsequently, the cell culture supernatant was collected for EV isolation and the cells were subjected to flow cytometric analysis.

Cell surface staining for flow cytometric analysis of THP-1 and EGI-1 human cell lines

Cell surface marker analysis was performed according to our previously published protocol.[30,31] Typically, 1.5 × 10^5 cells were suspended in FcR blocking reagent (Miltenyi Biotec, Bergisch Gladbach, Germany). Anti-CD133/2 antibodies (130-112-195, Miltenyi Biotec) and anti-CD44v6 antibodies (130-111-238, Miltenyi Biotec) were added and titrated against their matching isotype (REA293 phycoerythrin, 130-107-771 and REA293 allophycocyanin, 130-113-446, Miltenyi Biotec) and used in concentrations according to the respective antibodies; 7-AAD (BD Pharmingen, NJ, USA, 559925) was used for dead cell exclusion. The cells were analyzed on a BD FACSCanto™ II system (BD Biosciences, Heidelberg, Germany). Antibody details are shown in Table S2. For technical validation of the anti-CD133/2 antibodies (clone 293C3), the extrahepatic CCA cell line EGI-1 was used as a positive control, whereas THP-1 cells served as a hematopoietic negative control, as previously described.[27,32]

Isolation of small EVs from human serum

Human serum (1 ml) was centrifuged at 10,000 × g for 10 min to remove lipids and most of the large EVs. EV-containing supernatant was made up to 2 ml with filtered PBS (pH 7.4; 0.22 μm). Diluted and precleared serum (2 ml) was loaded onto a qEV2/70 size exclusion chromatography (SEC) column, and a buffer flow volume of 14.1 ml was discarded, after which the following five fractions (2 ml each) were collected, pooled, and concentrated to 1 ml of a large EV-depleted small EV fraction with a 3-kDa MWCO filter for the ExoView® Reader-based study. We reported this SOP previously[30] and the isolated small EVs were characterized by nanoparticle tracking analysis and Western electrophoresis (WES) in compliance with the Minimal Information for Studies of Extracellular Vesicles (MISEV) 2018 guidelines.[33,34] Given that the SOP has remained unchanged, it is adequate to reference our previous publication in alignment with MISEV.

Nanoparticle tracking analysis for the determination of size distribution and particle count

The particle diameter and concentration were measured by using a ZetaView instrument (Particle Metrix, Inning am Ammersee, Germany). The SOP was set to a laser wavelength of 488 nm, in 11 positions, with a sensitivity of 79.2, framerate of 30, shutter of 70, minimum brightness of 30, maximum area of 1,000, minimum area of 5, a trace length of 15, and 30 nm/class. The particles/ml, the peak particles/ml, and the peak particle diameter were automatically calculated by the ZetaView software (Version 8.05.14. SP7, Particle Metrix).

SP-IRIS for EV antigen profiling

ExoView® R100 (Unchained Labs, Boston, MA, USA) was used for interferometric and fluorescence imaging of individual small EVs immobilized on the surface of a microchip. Each chip, comprising a silicon/silicon dioxide double layer, is covered with a microarray of four individual functionalized antibody spots in technical triplicates, allowing for multiplexed EV capture and detection. Human ExoView® Tetraspanin Chips were supplied precoated with anti-CD9, anti-CD63, and anti-CD81 EV capture antibodies and a mouse (M)IgG control (Fig. 1). Before sample acquisition, the custom antibody concentrations were titrated in the recommended range of 0.1 to 10 μg/ml. A concentration of 1 μg/ml was found to provide the optimal results. Following the ExoView® kit assay protocol (v380.6, revised August 2021), precoated human ExoView® Tetraspanin Chips were prescanned with ExoView Scanner software (Version 3.2, NanoView Biosciences, Brighton, MA, USA) to detect debris before loading samples. Small EV samples were diluted in 1 × incubation solution I and incubated on the chips at room temperature for 18 h, allowing the EVs to become immobilized on the predetermined capture spots. After incubation, unbound particles were washed away by washing the chips three times at 500 × *g* for 3 mn in 1 × solution A on a rocking platform, followed by immunolabeling of captured small EVs with CD133/2 phycoerythrin in blocking

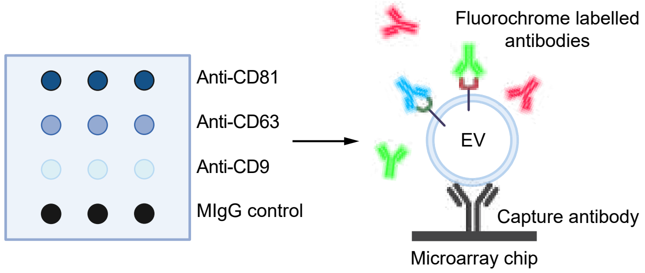

Fig. 1. Principle of EV detection by SP-IRIS on the ExoView® R100 platform. eft) A precoated microarray chip with triplicate spots of the capture antibodies anti-CD9, anti-CD63 and anti-CD81, which are specific to proteins typically found on small EVs and, thus, are used to capture small EVs on the chip surface. An MIgG control was used to determine unspecific binding. (Right) EVs are captured on the surface of the chip on the capture antibodies. Unbound EVs are washed away. EVs are subsequently stained with up to three different fluorochrome-labeled antibodies. Unbound antibodies are washed away and immunolabeled EVs are imaged in white light (size) and three different fluorescent channels. Created with BioRender (BioRender.com). EV, extracellular vesicle; MIgG, mouse IgG; SP-IRIS, single-particle interferometric reflectance imaging sensor.

solution at room temperature at 500 x *g* for 1 h, which were then measured. The resulting data were analyzed using an ExoView® Analyzer (Version 3.2, formerly NanoView Biosciences).

Code and analysis workflow

All data-driven analyses were implemented in Python (CPython 3.11.5; Python Software Foundation, Wilmington, DE, USA) within a reproducible Google Colab environment (Google Colaboratory, Mountain View, CA, USA). The full, timestamped analysis Google Colab notebook file is provided in the supplemental data. Software libraries (pandas 2.2, numpy 1.26.4, scikit-learn 1.6.1, matplotlib 3.10.0, seaborn 0.13.2, scipy 1.14.1, and lifelines 0.30.0) are explicitly documented to ensure computational reproducibility fulfilling Standards for Reporting Diagnostic Accuracy–Artificial Intelligence extension (STARD-AI) recommendations for transparency. All analyses were executed in Google Colab (standard CPU runtime; typical configuration: Intel Xeon 2.2 GHz, 16 GB RAM) without GPU acceleration.

In brief, the study CSV was loaded and continuous variables were standardized (z-score) before model fitting. The dataset was split into training and test sets (80:20, random_state = 42) for hybrid model development. Missing continuous biomarker values were imputed using disease-stratified median imputation. Importantly, internal robustness was evaluated on a dataset with high overall completeness and fully complete EV measurements, reducing the likelihood that observed stability reflected artefacts of missing data handling. Table S3 documents missing values, showing that these were limited, nonsystematic, and confined primarily to routine serological parameters, whereas all EV-derived markers were complete across both cohorts.

This analytic pipeline represents a supervised machine-learning (ML) workflow, in which least absolute shrinkage and selection operator (LASSO) logistic regression was trained on labeled diagnostic outcomes (HCC = 0, iCCA = 1) to identify discriminative biomarkers. PCA was subsequently applied in an unsupervised manner to visualize variance structure and confirm that the selected features contributed to disease separation. All random seeds were fixed, and model training followed a prespecified pipeline to prevent information leakage between training and test data. PCA was used as an exploratory visualization and to summarize variance structure; it did not use the test set to select features or thresholds.

Marker selection took advantage of two complementary approaches: (1) a logistic least absolute shrinkage and selection operator (LASSO; LogisticRegression with L1 penalty, solver = ‘liblinear’) identified candidate predictors for classification; and (2) PCA (n_components = 4) quantified the contribution of each marker by summing absolute loadings across the first four principal components (PCs). LASSO coefficients and PCA contributions were merged to define a final data-driven marker set. For visualization of regularization behavior, we produced a LASSO path using LassoCV. As reflected in the accompanying Python analysis workflow, LASSO and PCA were applied independently to the same feature set as complementary, reproducible exploratory tools, and their outputs were jointly considered to prioritize markers based on consistency, interpretability, and translational feasibility.

For diagnostic model discovery, we trained logistic regression hybrid models on all non-empty EV and serologic subsets and evaluated discrimination by AUROC. AUROC *p* values and comparisons were computed using a DeLong implementation applied to model-predicted probabilities. For the additive scoring systems, optimal cut-offs for each biomarker were derived from ROC analysis using Youden’s index; in a planned alternative variant, that of AFP was dichotomized using a fixed cut-off of 20 ng/ml, consistent with recommendations from the AASLD.[35] In the additive score, each biomarker above its threshold contributed 1 point, and the total score (sum of points) was used for classification. The simplified PRISM-based version was designed for end users without computational expertise, allowing manual scoring in clinical practice.

Importantly, the dataset was split into training and held-out test sets before any model-building steps. All feature selection (LASSO), model fitting, and threshold derivations (including ROC/Youden cut-offs for individual biomarkers and the optimal total-score threshold for the additive scoring system) were performed using the training set only. The held-out test set was not used for feature selection or threshold optimization and was used only for final performance estimation. Furthermore, the additive scoring systems were derived from the selected features of the hybrid model and did not result from an exhaustive combinatorial search across multiple arbitrary model configurations.

Statistical analysis

Most analyses were conducted in Python (CPython 3.11.5; Python Software Foundation) using scikit-learn, scipy, and statsmodels. Normality was assessed by the Shapiro–Wilk test. Whereas albumin and thrombocytes showed approximate normal distribution, most biomarkers, including AFP, CA19-9, CRP, and all EV-derived variables, displayed right-skewed distributions. Consequently, Welch’s *t* test was used for group comparisons, because it remains robust to unequal variances and moderate deviations from normality. For heavily skewed variables, results were cross-checked using Mann-Whitney *U* tests, yielding consistent significance patterns.

Diagnostic model evaluation used AUROC as the primary metric. AUROC significance and pairwise AUROC comparisons were assessed with DeLong’s test. Optimal thresholds were defined by Youden’s index unless otherwise specified. Sensitivity, specificity, positive predictive values (PPVs) and negative predictive values (NPV)s were calculated from 2×2 contingency tables at the chosen threshold and statistical significance of classification performance was assessed by χ^2 tests.

Post-hoc effect size (Cohen’s d) and statistical power were computed from the Welch’s t tests to assess sensitivity of biomarker comparisons (Fig. S1). Model robustness and uncertainty were quantified by: (1) nonparametric bootstrap resampling (1,000 iterations) for AUROC CIs; and (2) Monte Carlo (MC) perturbation analysis (±5%, ±10%, and ±20% biomarker noise, 500 iterations each) to evaluate stability under analytical variability. All random seeds were fixed for reproducibility. To further assess model stability and address potential split dependency in this small cohort, we performed 200 repeated random 80:20 train–test splits. In each iteration, LASSO-based feature selection and model derivation were repeated from scratch on the

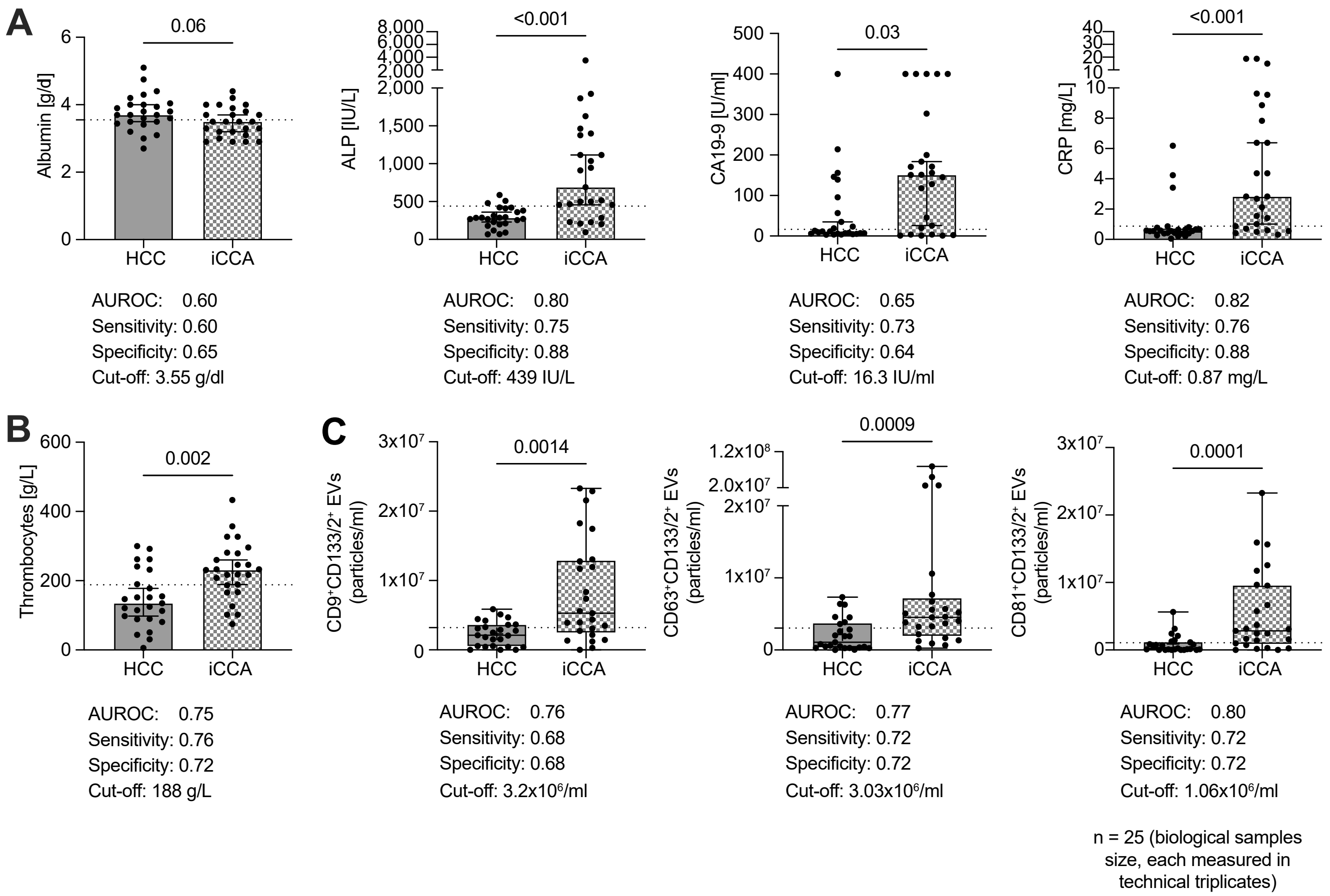

Fig. 2. Serological parameters and CD133/2$^+$ sEVs in HCC and iCCA. (A) Overview of routinely assessed standard serum parameters in biliary and hepatic malignancies, including albumin, ALP, CA19-9, and CRP.(B) Platelet (thrombocyte) counts. (C) Total numbers of CD133/2$^+$ sEVs and their CD9$^+$, CD63$^+$, and CD81$^+$ subpopulations immobilized on Human ExoView® Tetraspanin Chips precoated with anti-CD9, anti-CD63, and anti-CD81 EV capture antibodies, including MIgG isotype controls. Bars represent median values with 95% CIs. Associated AUROC values, sensitivity, specificity, and cut-off values were calculated using GraphPad PRISM and subsequently applied in the analogous additive scoring system. Statistical analyses were performed using GraphPad PRISM (version 10.4.1). Group comparisons were conducted using two-sided Mann-Whitney *U* tests because of non-normal data distribution. Exact *p* values are shown and were as follows: albumin, $p = 0.06$; ALP, $p < 0.001$; CA19-9, $p = 0.03$; and CRP, $p < 0.001$ (A); thrombocytes, $p = 0.002$ (B); CD9$^+$CD133/2$^+$, $p = 0.0014$; CD63$^+$CD133/2$^+$, $p = 0.0009$; and CD81$^+$CD133/2$^+$, $p = 0.0001$ (C). Post-hoc effect size (Cohen's d) and power analyses were derived from the Mann-Whitney *U* test results to quantify the discriminatory sensitivity of each biomarker comparison (Fig. S1). ALP, alkaline phosphatase; CRP, C-reactive protein; EV, extracellular vesicle; HCC, hepatocellular carcinoma; iCCA, intrahepatic cholangiocarcinoma; MIgG, mouse IgG; sEV, small extracellular vesicle.

training set, followed by evaluation on the independent test set. For each split, test AUROC and selected feature sets were recorded. The distribution of AUROCs and feature selection frequencies were summarized descriptively.

Overall survival (OS) was assessed primarily in GraphPad PRISM v10.4.1 (GraphPad Software Inc., San Diego, CA, USA) using Kaplan–Meier estimation and the Mantel–Cox (log-rank) test; the Gehan–Breslow–Wilcoxon test is reported as a sensitivity analysis because it weights early events more heavily. Median survival times are reported with 95% CIs. Group comparisons by dichotomized biomarker levels (above *vs*. at or below the median) were tested with the log-rank test and corresponding hazard ratios (HRs) with 95% CIs were estimated using Cox proportional hazards regression (exploratory analyses performed with the lifelines package; Schoenfeld residuals were examined to assess proportional hazards). All survival analyses are exploratory given the sample size.

All analyses complied with STARD 2023 recommendations for diagnostic accuracy studies to ensure transparency and reproducibility.[36]

Results

Detection of CD133/2 and CD44v6 on cancer cell line EGI-1

Anti-CD133/2 antibodies established on the extrahepatic CCA cell lines EGI-1 and THP-1 served as a control.[27,32] EGI-1 showed a higher percentage of CD133/2$^+$ cells compared with THP-1 independently of whether they were stimulated or not, serving as natural CD133/2 positive control (Fig. S2).

Serological standard parameters in patients with HCC and iCCA

In total, 50 patients classified as LR-M were included in our study, comprising 25 with iCCA and 25 with HCC. LR-M

lesions account for <10% of primary liver cancer cases, yet they represent the single most diagnostically ambiguous LI-RADS category.[37] A selection of routinely taken serological parameters were investigated in HCC and iCCA, including AFP, albumin, ALP, CA19-9, CRP, and thrombocytes. AFP was not conclusive measured in HCC and iCCA (Table S1). Albumin showed a trend toward higher levels in HCC compared with iCCA (AUROC = 0.60, sensitivity = 60%, specificity = 65%); however, this difference did not reach statistical significance (p = 0.06; Fig. 2A). Median ALP was 2.4-fold elevated in iCCA (AUROC = 0.80, sensitivity = 75%, specificity = 88%; p <0.001, Fig. 2A). The known pan-tumor marker CA19-9 was strongly and significantly increased in iCCA compared with HCC (AUROC = 0.65, sensitivity = 73%, and specificity = 64%; p = 0.03; Fig. 2A). The median of the inflammatory marker CRP was significantly elevated in iCCA by 5.1-fold (AUROC = 0.82, sensitivity = 76%, and specificity = 88%; p <0.001; Fig. 2A). Thrombocytes (platelets) were only slightly increased in iCCA (AUROC = 0.75, sensitivity = 76%, and specificity = 72%; p = 0.002; Fig. 2B). In sum, these six routinely measured parameters did not exceed a sensitivity of 82% and a specificity of 0.88% in our cohorts.

Detection of CD133/2 and CD44v6 on human blood-borne small EVs

HCC and iCCA samples differed significantly in their small EV subpopulations CD9^{+}CD133/2^{+} (p = 0.0014) and CD63^{+}CD133/2^{+} (p = 0.0009) and CD81^{+}CD133/2^{+} (p = 0.0001; Fig. 2C). To assess the diagnostic potential of CD9^{+}CD133/2^{+}, CD63^{+}CD133/2^{+}, and CD81^{+}CD133/2^{+} small EV subpopulations, their data were subjected to a ROC curve analysis with a 95% CI. The CD9^{+}CD133/2^{+} small EV median was significantly elevated by 2.5-fold (AUROC = 0.76 and a sensitivity = 68% and a specificity = 68%). The CD63^{+}CD133/2^{+} small EV median was significantly increased by 4.2-fold (AUROC = 0.77, sensitivity = 72%, and specificity = 72%). The CD81^{+}CD133/2^{+} small EV median was significantly elevated 12.2-fold (AUROC = 0.80, calculated sensitivity = 72%, and specificity = 72%). CD44v6^{+} small EVs and their subpopulations were below detection levels and could not be measured (data not shown).

Exploratory analysis supporting marker prioritization

To identify informative biomarkers for differentiating HCC from iCCA, exploratory analyses were applied to the full feature set using complementary supervised and unsupervised perspectives. LASSO regression was used to assess feature stability under regularization. The LASSO path plot (Fig. 3A) showed that ALP, CRP, and CD9^{+}CD133/2^{+} small EVs entered the model early with relatively stable coefficients, whereas thrombocytes, AFP, albumin, and CD81^{+}CD133/2^{+} small EVs showed smaller or later contributions. CA19-9 and CD63^{+}CD133/2^{+} small EVs entered late with comparatively low coefficients.

In parallel, PCA was used to explore the variance structure of the dataset by summing absolute loadings across the first four PCs. The highest cumulative contributions were observed for CRP (1.5117), AFP (1.2465), CD63^{+}CD133/2^{+} small EVs (1.1894), thrombocytes (1.0554), and CA19-9 (1.0331), followed by CD9^{+}CD133/2^{+} small EVs, ALP, albumin, and CD81^{+}CD133/2^{+} small EVs. The PCA biplot (Fig. 3B) illustrates the distribution of markers along PC1 and PC2, highlighting distinct variance contributions among serological parameters and EV subpopulations. Joint interpretation of these analyses showed that CRP exhibited both a high contribution to variance and a robust positive standardized LASSO coefficient (0.8163), indicating consistent relevance across analytical perspectives. By contrast, AFP demonstrated a substantial variance contribution but a negative standardized LASSO coefficient (-0.4900), reflecting an inverse association within the fitted model. Importantly, LASSO coefficients reflect relative importance within the fitted model and do not imply independent or causal predictive strength, particularly in the presence of correlated features.

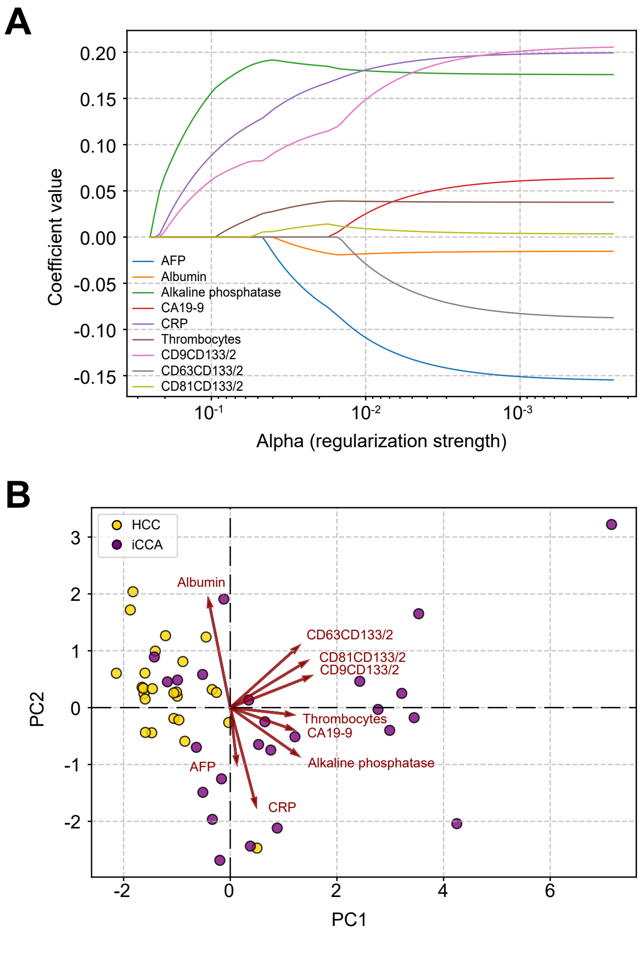

Fig. 3. Selection of the final marker set using LASSO and PCA. (A) The LASSO path plot illustrates the selection process of candidate markers using LASSO regression first. Early entrants, such as ALP, CRP, and CD9^{+}CD133/2^{+} small EVs, achieved high coefficient values, indicating consistent relevance within the fitted model. By contrast, thrombocytes, AFP, and albumin contributed with lower coefficients, whereas late entrants, including CA19-9 and CD63^{+}CD133/2^{+} small EVs, had the weakest contributions. (B) PCA biplot: PCA was applied to the full feature set to visualize marker distribution along the first two PCs (PC1 and PC2). The biplot depicts how the investigated markers cluster in distinct regions, with CRP, AFP, and thrombocytes forming separate groups from CA19-9 and EV subpopulations. AFP, alpha-fetoprotein; ALP, alkaline phosphatase; CRP, C-reactive protein; EV, extracellular vesicle; PC, principal component.

Based on this convergent evaluation, two logistic regression–based models were defined for further performance assessment**:** (1) hybrid model with AFP: included AFP, CA19-9, $CD9^{+}CD133/2^{+}$ small EVs, $CD81^{+}CD133/2^{+}$ small EVs, CRP, and ALP. This model achieved an AUROC of 0.857, a sensitivity of 0.71, a specificity of 1.00, and p = 0.050 (Table S4); and (2) hybrid model without AFP: excluded AFP, comprised CA19-9, $CD9^{+}CD133/2^{+}$ small EVs, $CD81^{+}CD133/2^{+}$ small EVs, CRP, and ALP, resulting in an AUROC of 0.905, sensitivity of 0.86, specificity of 1.00, and p = 0.050 (Table S4; Fig. 3).

Internal robustness across repeated random splits

Across 200 repeated random train–test splits (Fig. S3A), the hybrid logistic model achieved a mean test AUROC of 0.82 (SD: 0.130; 95% CI: 0.800–0.837; median: 0.84, IQR: 0.76–0.92). The additive score achieved a mean test AUROC of 0.86 (SD: 0.100; 95% CI: 0.845–0.873; median: 0.86, IQR: 0.80–0.94). The additive score outperformed the hybrid model consistently across splits (paired Wilcoxon signed-rank $p = 8.879 \times 10^{-10}$; paired t test $p = 7.420 \times 10^{-10}$). Core markers were also selected with high frequency (CRP 100%, ALP 97.5%, thrombocytes 94.0%, $CD9^{+}CD133/2^{+}$ EVs 93.0%, and $CD81^{+}CD133/2^{+}$ EVs 87.0%), whereas AFP (32.5%) and $CD63^{+}CD133/2^{+}$ EVs (2.5%) were selected less consistently (Fig. S3B). Core markers demonstrated consistently high selection frequencies across splits, supporting the stability of the core biomarker panel, whereas AFP and $CD63^{+}CD133/2^{+}$ EVs showed limited and inconsistent selection.

Additive scoring system development

To translate the LASSO/PCA-derived hybrid models into a clinically applicable paper-pencil format, we developed an additive scoring system. For each selected biomarker, an optimal diagnostic cut-off was determined by ROC analysis using Youden's index. In one variant, the AFP cut-off was fixed at 20 ng/ml according to AASLD guidelines. Each marker value exceeding its cut-off was assigned 1 point (favoring iCCA), whereas values below the threshold scored 0 points (favoring HCC).

The sum of all marker points yielded a total score used for classification: (1) 0–3 points: classified as HCC; and (2) 4+ points: classified as iCCA.

Then, the additive scoring system was evaluated in two versions based on the hybrid models: (1) additive hybrid model with AFP: all selected markers, including AFP, use ROC–Youden-derived optimal cut-offs; and (2) additive hybrid model without AFP: AFP is excluded from the scoring system (Fig. 4).

Both additive hybrid models demonstrated strong diagnostic accuracy, with AUROC >0.95 and high sensitivity combined with perfect specificity (Table S5).

Supplementary raw data cut-offs

All marker-specific cut-off values used for classification are summarized in Table 1. Cut-offs for both LASSO/PCA-derived hybrid models were determined using ROC analysis and Youden's index. In the fixed AFP model, the threshold of AFP was set to 20 ng/ml, following the AASLD guideline.[35] The PRISM-based 5-point scoring system applies literature- and PRISM-derived thresholds, enabling an analogous yet computationally independent clinical approach (Fig.5).

Comparison of additive scoring systems using different threshold definitions

To evaluate the stability and transferability of the hybrid additive scoring approach, we compared four additive models that differed only in the source of their biomarker cut-off values while applying the same binarized scoring logic. The hybrid models (rows 1–3, Table 2) were based on AUROC-derived Youden thresholds identified within the LASSO/PCA hybrid framework, including AFP, excluding AFP, or integrating a fixed AFP cut-off (20 ng/ml) in accordance with AASLD guidelines. By contrast, the 5-point scoring system (PRISM) (row 4, Table 2) used calculated cut-offs for ALP, CRP, CA19-9, $CD9^{+}CD133/2^{+}$, and $CD81^{+}CD133/2^{+}$, as established in PRISM analyses, thereby providing an external, model-independent analog. All four additive models achieved high diagnostic accuracy (AUROC 0.90–0.93, Table 2), confirming the robustness of the combined serological + EV marker constellation across both data-driven and literature-based thresholds. Importantly, the PRISM-based 5-point scoring system performed only slightly below the ML-derived fully data-driven hybrid models, yet can be readily applied in clinical practice without any specialized software or computational tools, making it a pragmatic and accessible option for diagnostic implementation.

Internal robustness and stability analysis (bootstrapping and MC simulation)

To assess the internal robustness of the PRISM-based 5-point scoring system, we conducted a MC simulation and bootstrapping analysis (Table S6). Bootstrapping with 1,000 iterations yielded a mean AUROC of 0.90 (95% CI: 0.807–0.968), confirming a stable diagnostic performance across random patient re-samplings. To further test resilience against analytical variability, we applied a MC perturbation procedure in which all biomarker values were randomly varied within predefined error ranges (±5%, ±10%, or ±20%) while keeping the model cut-offs constant. Each noise level was simulated 500 times, and the AUROC was recalculated for every perturbed dataset. The resulting mean AUROCs remained consistently high (0.89, 0.89, and 0.89 for ±5%, ±10%, and ±20%, respectively) with narrow SDs (≤0.015) and no drop below 0.84 even under ±20% variability (Table S6). These results demonstrate that the PRISM-based additive scoring model retains its discriminative accuracy even under realistic levels of measurement noise, highlighting its robustness and practical reliability for clinical implementation. Bootstrap-derived percentile intervals for cohort-derived ROC–Youden cut-offs are shown in Table S7.

Survival analysis results

To further assess the clinical relevance of CD133/2-positive small EV subpopulations, OS was analyzed by the Kaplan–Meier method with two-sided log-rank testing. Across disease types, patients with iCCA demonstrated significantly

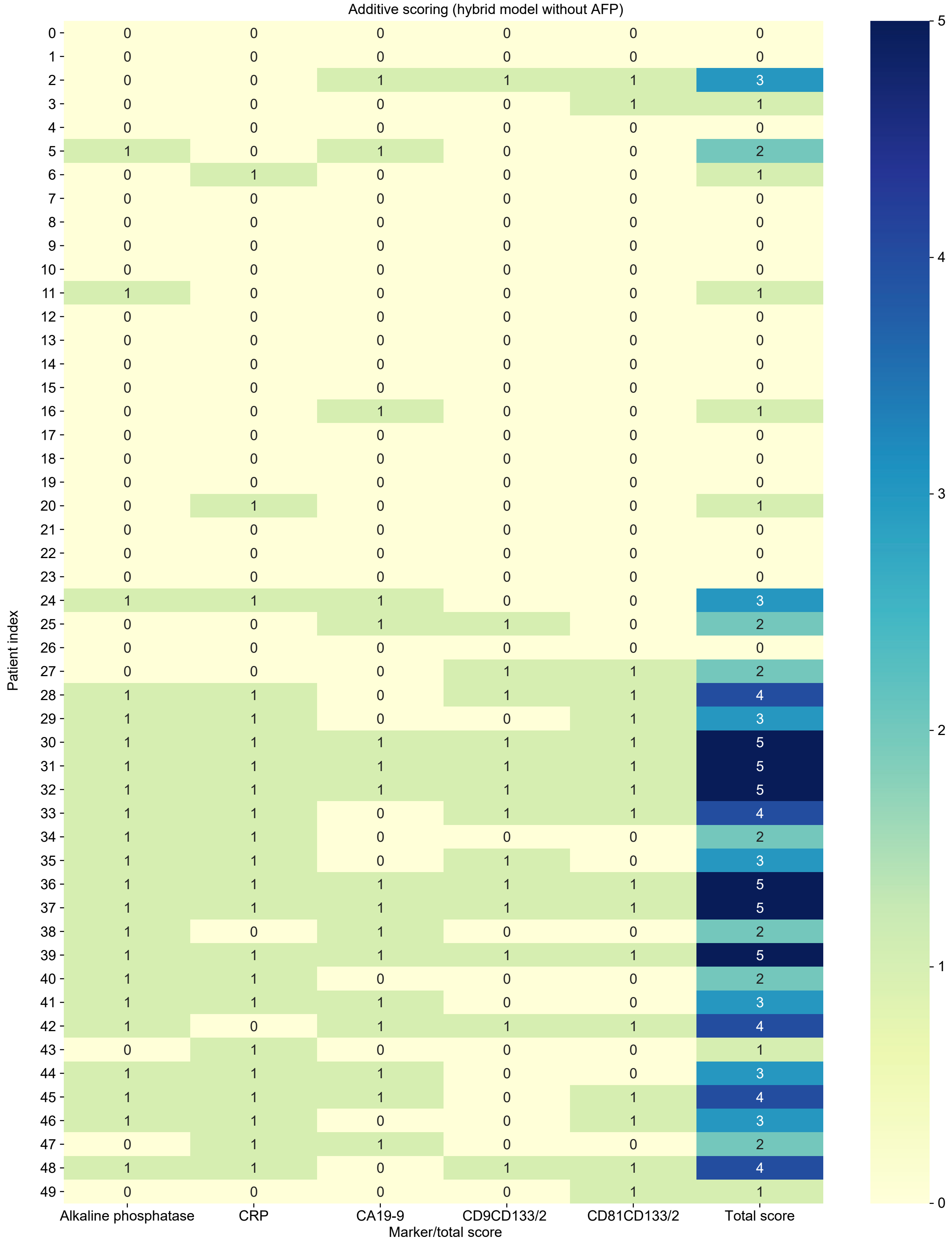

Fig. 4. Additive scoring results based on the hybrid model without AFP of each included patient with HCC and iCCA and the total score. As defined by us, 0–3 points was classified as HCC, and 4 or 5 points was classified as iCCA for LR-M patients. Depicted are the Patient Index (left), marker (bottom), and heatmap color-code (right). HCC rows 0–24 and iCCA rows 25–49. AFP, alpha-fetoprotein; HCC, hepatocellular carcinoma; iCCA, intrahepatic cholangiocarcinoma.

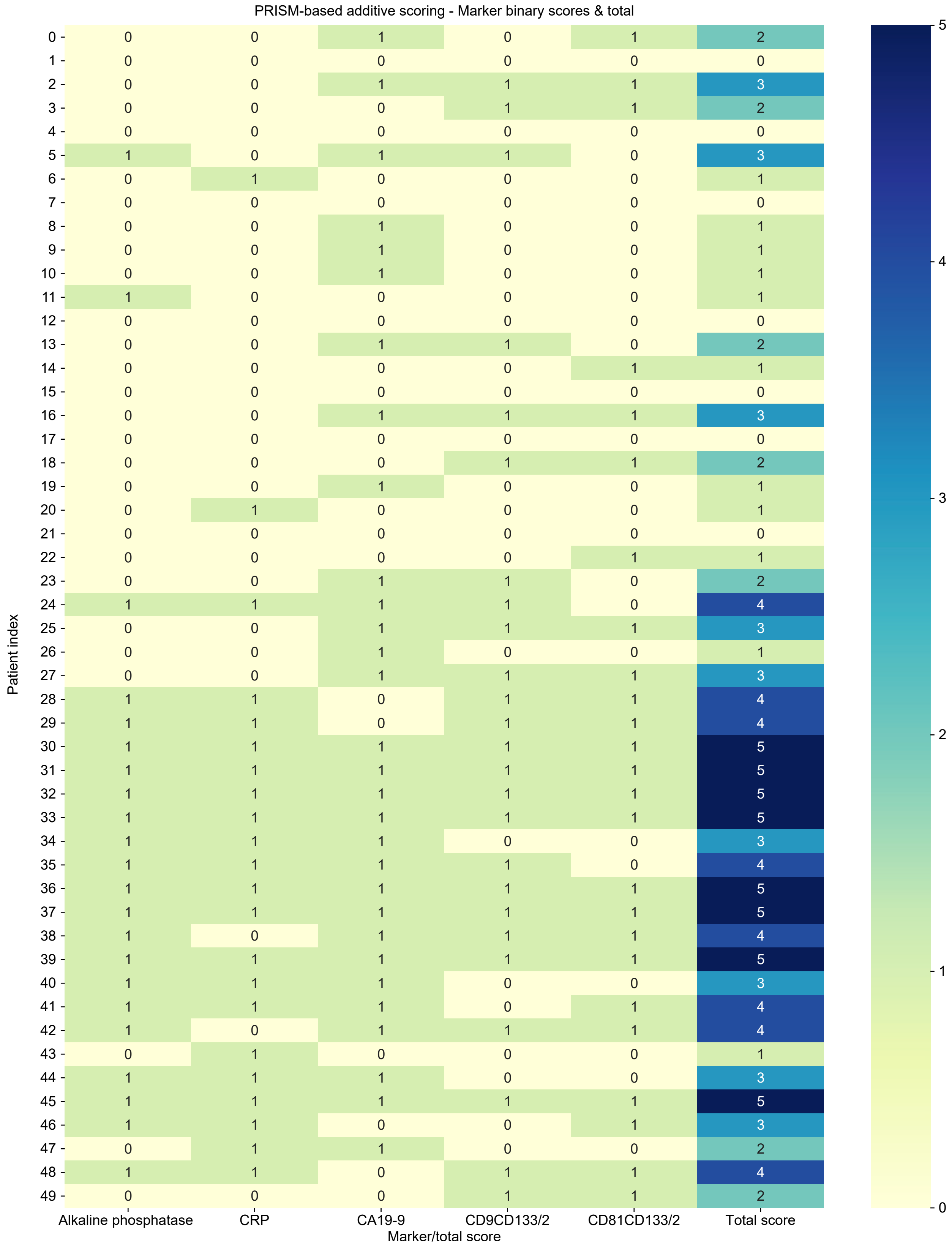

Fig. 5. Five-point scoring results based on PRISM-derived cut-off values without AFP of each included patient with HCC and iCCA and the total score. As defined by us, 0–3 points was classified as HCC, and 4 or 5 points was classified as iCCA for LR-M patients. Depicted are the Patient Index (left), marker (bottom), and heatmap color-code (right). HCC rows 0–24 and iCCA rows 25–49. AFP, alpha-fetoprotein; HCC, hepatocellular carcinoma; iCCA, intrahepatic cholangiocarcinoma.

Table 1. Marker-specific cut-offs derived for different modeling strategies.

Model	Marker	Cut-off
Hybrid model with AFP (ROC–Youden)	AFP	400 ng/ml
	ALP	456 U/L
	CA19-9	117 U/ml
	CRP	0.88 mg/dl
	$CD9^+CD133/2^+$ EVs	5.292×10^6/ml
	$CD81^+CD133/2^+$ EVs	2.45055×10^6/ml
Hybrid model without AFP (ROC–Youden)	ALP	456 U/L
	CA19-9	117.9 U/ml
	CRP	0.88 mg/dl
	$CD9^+CD133/2^+$ EVs	5.292×10^6/ml
	$CD81^+CD133/2^+$ EVs	2.45055×10^6/ml
5-point scoring system without AFP (PRISM)	ALP	439 U/L
	CA19-9	16.3 U/ml
	CRP	0.87 mg/dl
	$CD9^+CD133/2^+$ EVs	3.2×10^6/ml
	$CD81^+CD133/2^+$ EVs	1.06×10^6/ml

ROC–Youden cut-offs for hybrid logistic regression models were optimized for discrimination of individual continuous predictors, whereas PRISM-based cut-offs were derived from single-marker ROC analyses and selected to support balanced contribution within a binary additive scoring system. Differences between cut-offs reflect these distinct optimization objectives. AFP, alpha-fetoprotein; ALP, alkaline phosphatase; CRP, C-reactive protein; EV, extracellular vesicle.

poorer survival compared with patients with HCC within the LR-M cohort (log-rank p = 0.005). In the iCCA subgroup (n = 25; above-median n = 13, ≤ median n = 12), patients with $CD9^+CD133/2^+$ small EV levels above the cohort median experienced substantially shorter OS compared with those with lower levels: median survival was 91.0 days *vs.* 389.5 days (median ratio: 4.28; 95% CI: 1.88–9.76). Survival differed significantly by log-rank (χ^2 = 7.829, p = 0.005) and by Gehan–Breslow–Wilcoxon testing (χ^2 = 5.324, p = 0.02). The log-rank (Cox) HR for death comparing above-median *vs.* ≤ median $CD9^+CD133/2^+$ levels was 2.80 (95% CI: 1.16–6.74) (Fig. 6A). In patients with HCC (n = 25; above-median n = 12, ≤ median n = 13), stratification by $CD81^+CD133/2^+$ showed shorter median survival in the above-median group (730 *vs.* 1,460 days). The log-rank test reached nominal significance (χ^2 = 4.488, p = 0.034; HR: 2.46, 95% CI: 0.996–6.06), whereas the Gehan–Breslow–Wilcoxon test did not (χ^2 = 3.048, p = 0.081), indicating that this observation should be interpreted cautiously (Fig. 6B). Proportional hazards assumptions were evaluated using Schoenfeld residual testing and showed no evidence of violation in any model (all p ≥0.337; Table S8). Other CD133/2-positive EV subpopulations and routine serological markers did not demonstrate consistent or statistically robust survival differences following median stratification. All survival analyses are exploratory.

Discussion

Rather than seeking a cancer-specific biomarker or an alternative screening tool for HCC, we aimed to determine whether integrated marker profiling could resolve the clinically unresolved entity assignment within LI-RADS LR-M lesions, distinguishing HCC from iCCA in cases where malignancy is established but imaging and standard serology remain nonspecific, a distinction with direct therapeutic and prognostic consequences.[13] To achieve this, we first isolated small EVs according to the latest MISEV guidelines[33] and characterized CD133-positive small EV subpopulations using SP-IRIS (ExoView® R100 platform), allowing us to differentially analyze $CD9^+CD133/2^+$, $CD63^+CD133/2^+$, and $CD81^+CD133/2^+$ small EVs. Initial AUROC analysis revealed that no single marker, whether EV based or serological, could independently achieve reliable diagnostic performance, because none reached an AUROC >0.85 (Fig. 3).

To prioritize a parsimonious and clinically interpretable biomarker set, we applied data-driven exploratory analyses to the full feature space. Rather than relying on a single statistical criterion, we evaluated marker relevance across complementary perspectives, including supervised discrimination performance and unsupervised variance structure. This approach confirmed that no individual serological or EV-derived marker achieved sufficient diagnostic accuracy alone, whereas a small constellation of markers consistently contributed to disease separation. Importantly, marker prioritization was guided not only by statistical signal strength, but also by biological plausibility, robustness, and translational feasibility. This strategy enabled us to distill a complex, high-dimensional dataset into a limited set of markers suitable for binarization and use in a paper-and-pencil additive scoring system. Importantly, our approach aligns with previous studies that have utilized LASSO regression for biomarker selection in high-dimensional genomic data, demonstrating its effectiveness in identifying key prognostic markers.[38,39] Similarly, the application of PCA in dimensionality reduction has been shown to capture the underlying variance structure in complex datasets, facilitating the identification of biologically relevant patterns.[40] By integrating these methodologies, our study enhances the robustness and clinical applicability of the biomarker panel.

Using the LASSO/PCA hybrid models as the core artificial intelligence (AI)/ML discovery framework, we next focused on translating these continuous models into clinically applicable scoring systems (Table 3). The hybrid models, developed with and without AFP, defined a stable, data-driven marker set integrating EV-derived and serological features, while also

Table 2. Diagnostic performance of the additive scoring approaches based on the indicated logic for differentiating iCCA from HCC in LR-M lesions.

Logic	AUROC	Optimal total score (Youden's Index)	Sensitivity	Specificity	χ^2 *p* value
Hybrid model with AFP	0.9264	2.0	0.88	0.88	<0.0001
Hybrid model without AFP	0.9240	2.0	0.88	0.88	<0.0001
Hybrid model with fixed AFP	0.9008	2.0	0.88	0.80	<0.0001
5-point scoring system (PRISM)	0.9064	3.0	0.84	0.84	<0.0001

AUROC values were computed in Python (scikit-learn) from ROC curves of the respective total scores. The 'Optimal Total Score' corresponds to the Youden-index threshold (maximum of TPR-FPR) derived from the ROC curve and used to dichotomize predictions (score ≥ threshold). Sensitivity and specificity were calculated from the resulting confusion matrix. Statistical significance was assessed using a two-sided Pearson χ^2 test applied to the 2 × 2 contingency table of predicted class versus ground truth (TP/FN/FP/TN; chi2_contingency). All models showed highly significant discrimination (χ^2 test, p <0.0001). AFP, alpha-fetoprotein; HCC, hepatocellular carcinoma; iCCA, intrahepatic cholangiocarcinoma.

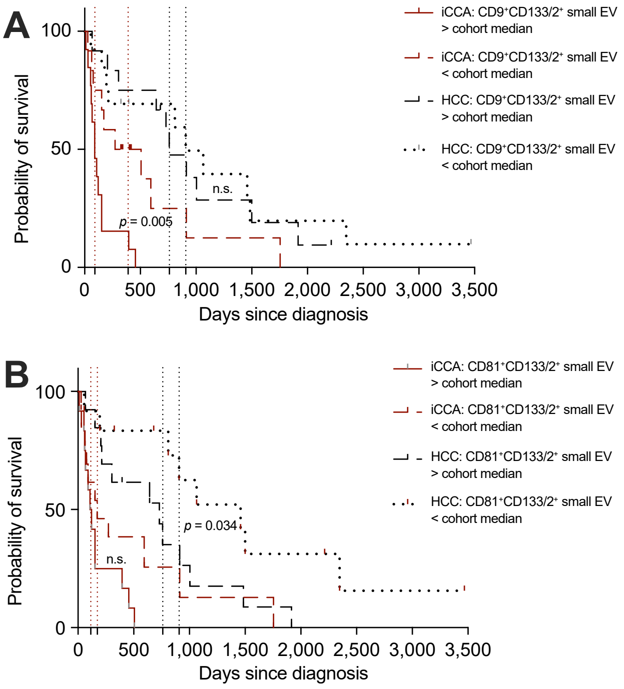

Fig. 6. Kaplan–Meier OS analysis in LR-M patients stratified by CD133/2 small EV subpopulations. Kaplan–Meier curves depict OS (days since diagnosis) stratified by above-median versus ≤ median marker levels within disease-specific subgroups (HCC and iCCA; each n = 25). 'Event' indicates death. All survival analyses are exploratory and tests were two sided. (A) $CD9^+CD133/2^+$ small EVs. Patients with iCCA (n = 25; deaths = 23; censored = 2) were stratified into above-median (n = 13) versus ≤ median (n = 12). Patients with above-median $CD9^+CD133/2^+$ EV levels showed shorter median OS (91.0 *vs.* 389.5 days; median ratio: 4.28, 95% CI: 1.877–9.761). Survival differed significantly by log-rank (Mantel–Cox) test (χ^2 = 7.829, p = 0.005) and by Gehan–Breslow–Wilcoxon test (χ^2 = 5.324, p = 0.020). The log-rank HR was 2.802 (95% CI:1.164–6.744) for above-median versus ≤ median groups. Patients with HCC (n = 25; deaths = 20; censored = 5) were stratified into above-median (n = 10) versus ≤ median (n = 15). Median OS was 760.0 *vs.* 907.0 days. No significant survival differences were observed (log-rank χ^2 = 0.119, p = 0.730; Gehan–Breslow χ^2 = 0.0077, p = 0.930). The log-rank HR was 1.162 (95% CI: 0.482–2.798). (B) $CD81^+CD133/2^+$ small EVs. Patients with iCCA patients (n = 25; deaths = 23; censored = 2) were stratified into above-median (n = 12) versus ≤ median (n = 13). Median OS was 114.5 *vs.* 173.0 days. Differences did not reach statistical significance (log-rank χ^2 = 2.840, p = 0.090; Gehan–Breslow χ^2 = 1.205, p = 0.270). The log-rank HR was 1.914 (95% CI: 0.818–4.476). Patients with HCC (n = 25; deaths = 20; censored = 5) were stratified into above-median (n = 12) versus ≤ median (n = 13). Median OS was 730.0 *vs.* 1460.0 days. Survival differed by log-rank test (χ^2 = 4.488, p = 0.034), whereas the Gehan–Breslow–Wilcoxon test did not reach significance (χ^2 = 3.048, p = 0.0808). The log-rank HR was 2.457 (95% CI: 0.996–6.064). Vertical dotted lines indicate the estimated time points at which survival reached 50% (median OS). EV, extracellular vesicle; HCC, hepatocellular carcinoma; HR, hazard ratio; iCCA, intrahepatic cholangiocarcinoma; OS, overall survival.

revealing that AFP, although selected by LASSO/PCA, did not improve overall discrimination and may introduce redundancy in this context. This observation is consistent with previous studies, where AFP alone was insufficient for diagnosis but improved classification when combined with other biomarkers, such as Dickkopf-1, Lectin-reactive AFP (AFP-3), and Des-gamma-Carboxy-Prothrombin (DCP), or as part of the GALAD score, which integrates AFP with additional markers.[41–43] These studies demonstrated that AFP is more effective when used in combination rather than as a standalone marker, reinforcing our findings that its diagnostic contribution is context dependent.

To enable practical clinical use, we implemented two additive representations of these hybrid models. First, additive scoring systems derived from the LASSO/PCA hybrid models were constructed by binarizing the selected markers using ROC–Youden-derived cut-offs, preserving data-driven marker prioritization while enabling manual score calculation. Second, we developed a fully simplified 5-point PRISM-based scoring system, applying independently derived PRISM cut-offs to the same marker set, resulting in an externally anchored, paper-and-pencil tool suitable for routine use without computational infrastructure. A PRISM-plus-AFP variant was evaluated to explore the effect of guideline-consistent AFP thresholds. Across all representations, diagnostic performance remained high, demonstrating that robust discrimination between HCC and iCCA in LR-M lesions is retained even when continuous ML models are distilled into simple threshold-based scoring systems.

Our additive scoring system was developed as a complementary tool to aid the differentiation of HCC and iCCA in LR-M patients, offering a minimally invasive liquid biopsy alternative to liver biopsy. Although liver biopsy remains the gold standard for histopathological confirmation in LR-M, it is an invasive procedure with known limitations, including sampling error, interobserver variability, and procedural risks.[44,45]

Notably, our hybrid models (AUROC 0.91–0.92) performed within the range of well-established diagnostic systems, such as LI-RADS (AUROC ~0.87–0.94)[46,47] and GALAD (AUROC ~0.93).[48] Whereas LI-RADS is primarily imaging based and GALAD relies on serological markers (AFP, AFP-L3, and DCP), our scoring system uniquely integrates EV-based markers with serology, creating a distinct advantage. Unlike LI-RADS, which mandates biopsy for LR-M cases, our approach provides a potential minimally invasive adjunct for stratification before biopsy, addressing an unmet clinical need. This supports its role as a complementary rather than a replacement tool, ensuring that patients at higher risk receive timely and appropriate interventions addressing a diagnostic space that is explicitly outside the intended scope of existing screening algorithms, such as GALAD.[49]

Our survival analysis revealed that $CD9^+CD133/2^+$ small EVs were the strongest prognostic signal, whereas $CD81^+CD133/2^+$ contributed more prominently to diagnostic separation in the hybrid models. A parsimonious explanation is that these EV subpopulations capture distinct biological states of the tumor ecosystem. CD9 is implicated in EV biogenesis, trafficking, and metastatic spread, supporting the notion that high $CD9^+CD133/2^+$ EV levels might mark aggressive, stem-like or dissemination-prone clones (metastatic competence and therapy tolerance).[50] By contrast, CD81 is tightly linked to cell–cell adhesion, and immune and stromal crosstalk, which might be more salient during earlier or locally confined disease and, therefore, enhance diagnostic discrimination without necessarily portending poor survival.[51] These functional roles are consistent with previous work positioning CD133 as a stemness/metastasis mediator in hepatobiliary cancers and tetraspanins (CD9/CD63/CD81) as organizers of EV cargo and target-cell interactions, together shaping invasion, immune evasion, and niche conditioning.[52,53]

Methodologically, the dominance of EV variables on PC1 (the major axis separating iCCA from HCC) supports a

systems-level EV signature. Activated or stressed tumor and stromal cells release more EVs; therefore, an elevated EV particle and/or cargo load could scale with tumor burden, intratumoral heterogeneity, or stemness programs. In iCCA, typically more heterogeneous than HCC,[54] this could amplify EV-borne signals (including CD133-bearing vesicles), aligning with: (1) PC1 loadings; (2) strong additive-model performance; and (3) the marked OS gap we observed for $CD9^{+}CD133/2^{+}$-high iCCA. Together, these patterns argue that EV phenotype is not merely correlative but likely informative of disease state and trajectory.

Given that the OS sample size was modest and the analyses were exploratory, these observations should be considered hypothesis generating; validation in larger, independent cohorts that include longitudinal and treatment-response data will be required to confirm whether $CD9^{+}CD133/2^{+}$ and $CD81^{+}CD133/2^{+}$ small EVs have distinct, clinically actionable diagnostic and prognostic utilities.

Despite the promising diagnostic performance of our model, some limitations should be acknowledged. One inherent bias of our study is the advanced disease stage observed in the iCCA group compared with HCC within the LR-M cohort. This reflects a well-known clinical reality rather than a selection bias. By definition, LR-M lesions require histological confirmation according to LI-RADS guidelines, leading to a delay in diagnosis. Moreover, unlike HCC, which benefits from established surveillance programs in patients with cirrhosis, iCCA is often diagnosed at more advanced stages because of its silent progression.[55] This imbalance in disease severity at baseline can influence some performance metrics, particularly in distinguishing LR-M iCCA from HCC. Of note, future studies with larger, prospectively recruited cohorts will be necessary to validate our pilot-findings and assess the robustness of the model across different clinical settings. Although repeated random splits demonstrated stable performance and feature selection, the absence of external validation remains a limitation and warrants prospective multicenter assessment.

Compared with traditional single-analyte approaches (AFP or CA19-9), additive scoring systems, both the LASSO/PCA-derived and the PRISM-based 5-point variants, integrate EV and serological dimensions and remain robust under bootstrap and MC perturbations. Practically, the PRISM-based 5-point score offers a paper-and-pencil pathway: fixed cut-offs, no software, and performance only marginally lower compared with the ML-optimized hybrid, making it attractive for routine workflows and external validation.

In conclusion, this pilot study demonstrates our thesis that a data-driven biomarker selection approach, integrating ML/AI and additive scoring, can significantly improve the differentiation of HCC and iCCA in LR-M patients. Hence, the proposed scoring system is designed for use in patients with LI-RADS LR-M lesions, where imaging confirms malignancy but does not allow reliable differentiation between HCC and iCCA, and where biopsy is currently recommended. The findings reinforce the diagnostic utility of small EV-based biomarkers while demonstrating that the role of AFP remains context dependent. Future validation in larger cohorts will be vital for assessing the real-world applicability of our approach, particularly in integrating EV-based markers with established serologic and imaging criteria.

Table 3. Overview of model architectures and scoring strategies developed for differentiation of iCCA from HCC in LR-M lesions.

Model	Marker selection	AFP	Approximate AUROC	Notes/Interpretation
LASSO/PCA hybrid (logistic regression, continuous)	Data-driven feature selection by LASSO/PCA	±AFP	0.857/0.905	Core AI/ML discovery model integrating serological + EV features; continuous variable space, not binarized
Additive scoring (LASSO/PCA hybrid)	Same LASSO/PCA-selected features; ROC–Youden-derived cut-offs	±AFP	0.962/0.952	Data-driven, binarized version of hybrid model; optimized thresholds enable near-perfect discrimination
5-point scoring system (PRISM)	Based on LASSO/PCA hybrid markers; PRISM-derived cut-offs	±AFP and PRISM only	0.926/0.924/0.906	Analogous, externally anchored model; slightly lower AUROC but readily usable in routine clinical practice without computational tools

Marker selection was performed using a combined LASSO and PCA framework. Approximate AUROC values are reported to illustrate relative model performance and were derived from ROC analyses of the respective models (see main text). This table is intended as a conceptual comparison of modeling strategies rather than a statistical hypothesis test; therefore, no formal significance testing between models was performed. AFP, alpha-fetoprotein; AI, artificial intelligence; EV, extracellular vesicle; ML, machine learning.

Clinical perspective

Our results provide a proof of concept that diagnostic and prognostic information can be extracted minimally invasively from the circulation in patients with radiologically indeterminate LR-M lesions. By integrating small EV signatures with routine serological markers, we extend the current LI-RADS workflow toward a liquid biopsy-based risk stratification approach. This strategy not only distinguishes HCC from iCCA with high accuracy in a pilot approach, but also mirrors patient survival, allowing early identification of biologically aggressive disease. In clinical practice, such an approach could reduce the need for invasive liver biopsy, guide allocation to curative versus palliative pathways, and accelerate decision-making in multidisciplinary tumor boards. Ultimately, it brings precision diagnostics closer to real-time, patient-centered hepatology.

Affiliations

[1]*Department of Internal Medicine I, University Hospital of the Rheinische Friedrich-Wilhelms-University, 53127 Bonn, Germany;* [2]*Institute of Molecular Medicine and Experimental Immunology, University Hospital of the Rheinische Friedrich-Wilhelms-University, 53127 Bonn, Germany;* [3]*Octavian Fodor Institute for Gastroenterology and Hepatology, Iuliu Haţieganu University of Medicine and Pharmacy, Cluj-Napoca, 400012, Romania;* [4]*Medical Department, Babeş-Bolyai University, Cluj-Napoca, 400162, Romania;* [5]*Department of General- Visceral- and Thoracic Surgery, German Armed Forces Central Hospital Koblenz, 56072 Koblenz, Germany;* [6]*Department of Integrated Oncology, Center for Integrated Oncology (CIO), University Hospital of Bonn, 53127 Bonn, Germany;* [7]*Department of Gastroenterology, Hepatology and Transplant Medicine, Medical Faculty, University of Duisburg-Essen, 45147 Essen, Germany;* [8]*Laboratory of Metabolic Liver Diseases, Department of General, Transplant and Liver Surgery, Centre for Preclinical Research, Medical University of Warsaw, Warsaw, Poland;* [9]*Center for Inflammation Research, Department of Anesthesia, Beth Israel Deaconess Medical Center, Harvard Medical School, Boston, MA, USA*

Abbreviations

AFP, alpha-fetoprotein; AI, artificial intelligence; AJCC, American Joint Committee on Cancer; ALP, alkaline phosphatase; BCLC, Barcelona Clinic Liver Cancer (classification system); CCA, cholangiocarcinoma; CRP, C-reactive protein; CT, computed tomography; DCP, des-gamma-carboxy prothrombin; EV, extracellular vesicle; HCC, hepatocellular carcinoma; HR, hazard ratio; iCCA, intrahepatic cholangiocarcinoma; LASSO, least absolute shrinkage and selection operator; LI-RADS, Liver Imaging Reporting and Data System; LR-M, LI-RADS category 'malignant, not specific for HCC'; MC, Monte Carlo; MIgG, mouse IgG; MISEV, Minimal Information for Studies of Extracellular Vesicles; ML, machine learning; MRI, magnetic resonance imaging; NPV, negative predictive value; OS, overall survival; PC, principal component; PPV, positive predictive value; SEC, size exclusion chromatography; sEV, small extracellular vesicle; SOP, standard operating procedure; SP-IRIS, single-particle interferometric reflectance imaging sensor; STARD-AI, Standards for Reporting Diagnostic Accuracy–Artificial Intelligence extension; WES, Western electrophoresis system.

Financial support

Studies were supported by the Deutsche Forschungsgemeinschaft (DFG, German Research Foundation) to MTK (DFG project number 410853455). VL-K is funded by the DFG under Germany's Excellence Strategy – EXC 2151 – 390873048 and DFG Project number 411345524 and 432325352. AGW. was funded by the German Armed Forces (Bundeswehr, KdoGesVersBw, project number 31K1–S-10 2023).

Conflict of interest

The authors declare no conflicts of interest.
Please refer to the accompanying ICMJE disclosure forms for further details.

Authors' contributions

EV Methodology, experimental EV data management and analysis, and experimental data interpretation: RJ-SS, VL-K, MTK. Provision of human samples and human data management: TM, AGW, AK, RS, EM, CG, LPM, RC, ZS, JB, HHS, MK, RS, AGW, IGHS-W, SCR. Funding acquisition: VL-K, RS, AGW, MTK. Writing – original draft: RJ-SS, MTK. Writing – review & editing: RJ-SS, TM, AGW, AK, RS, EM, CG, LPM, RC, ZS, JB, HHS, MK, IGHS-W, SCR, VL-K, MTK. Original study design and project supervision: MTK.

Data availability

All shared materials will include well-documented Python notebooks (Google Colab compatible) and are provided in the supplemental data.

Acknowledgements

This article is based on work from the European Network for the Study of Cholangiocarcinoma and the COST Action CA22125 Precision medicine in biliary tract cancer (Precision-BTC-Network) supported by COST (European Cooperation in Science and Technology: www.cost.eu) to MK and VL-K. This study was reported as a poster talk at The Liver Meeting, Boston, MA, USA, 2023, Abstract 98: 'A novel 5-point scoring system for the differentiation of HCC from intrahepatic CCA in LR-M patients', Session: Novel Diagnostics in Liver Cancer.

STARD-AI compliance statement

This study adheres to the core principles of the STARD-AI 2025 guidelines for transparent reporting of AI-driven diagnostic accuracy studies. All model steps, from preprocessing to validation, were prespecified and reproducible (random seeds fixed and Colab code archived). Participant eligibility, reference standards, and index test procedures were fully defined, with balanced disease groups (25 HCC and 25 iCCA). The ML components (LASSO/PCA Hybrid and PRISM-based additive models) were explicitly described, including input features, output variables, and performance metrics (AUROC, sensitivity, specificity, PPV, and NPV). Model robustness was assessed via 1,000-fold bootstrap and MC perturbation analyses. To support clinical interpretability, the PRISM-based 5-point system was designed for use by clinicians without computational expertise. Given the limited sample size, algorithmic bias assessment was restricted to verification of balanced sex and disease distribution. Anonymized datasets and analysis code are available from the corresponding author upon reasonable request.

Declaration of generative AI and AI-assisted technologies in the manuscript preparation process

During the preparation of this work, the authors used ChatGPT (OpenAI) as an AI-assisted language tool to support editing, rephrasing, and improving readability. No AI tools were used for data analysis, statistical calculations, figure generation, or result interpretation.
All content generated with AI assistance was critically reviewed, edited, and validated by the authors, who take full responsibility for the accuracy, integrity, and originality of the published work.

Supplementary data

Supplementary data to this article can be found online at https://doi.org/10.1016/j.jhepr.2026.101826.

References

Author names in bold designate shared co-first authorship

[1] Ferlay J, Colombet M, Soerjomataram I, et al. Cancer statistics for the year 2020: an overview. Int J Cancer 2021;149:778–789.
[2] Llovet JM, Zucman-Rossi J, Pikarsky E, et al. Hepatocellular carcinoma. Nat Rev Dis Primers 2016;2:16018.
[3] Siegel RL, Kratzer TB, Wagle NS, et al. Cancer statistics, 2026. CA Cancer J Clin 2026;76:e70043.

[4] Forner A, Llovet JM, Bruix J. Hepatocellular carcinoma. Lancet 2012;379:1245–1255.
[5] Gomaa AI, Khan SA, Leen EL, et al. Diagnosis of hepatocellular carcinoma. World J Gastroenterol 2009;15:1301–1314.
[6] Patel T. Clinical diagnosis and management of intrahepatic cholangiocarcinoma. Clin Liver Dis 2014;3:56–59.
[7] Singal AG, Pillai A, Tiro J. Early detection, curative treatment, and survival rates for hepatocellular carcinoma surveillance in patients with cirrhosis: a meta-analysis. Plos Med 2014;11:e1001624.
[8] Bridgewater J, Galle PR, Khan SA, et al. Guidelines for the diagnosis and management of intrahepatic cholangiocarcinoma. J Hepatol 2014;60: 1268–1289.
[9] Roskams TA, Libbrecht L, Desmet VJ. Progenitor cells in diseased human liver. Semin Liver Dis 2003;23:385–396.
[10] Tanimizu N, Nakamura Y, Ichinohe N, et al. Hepatic biliary epithelial cells acquire epithelial integrity but lose plasticity to differentiate into hepatocytes in vitro during development. J Cell Sci 2013;126:5239–5246.
[11] Lozano RNM, Foreman K. Global and regional mortality from 235 causes of death for 20 age groups in 1990 and 2010: a systematic analysis for the Global Burden of Disease Study. Lancet 2012;380:2095–2128.
[12] World Health Organization. WHO classification of tumours of the digestive system. Lyon: IARC; 2010.
[13] Lee YT, Wang JJ, Zhu Y, et al. Diagnostic criteria and LI-RADS for hepatocellular carcinoma. Clin Liver Dis 2021;17:409–413.
[14] Wolf P. The nature and significance of platelet products in human plasma. Br J Haematol 1967;13:269–288.
[15] Skog J, Würdinger T, van Rijn S, et al. Glioblastoma microvesicles transport RNA and proteins that promote tumour growth and provide diagnostic biomarkers. Nat Cell Biol 2008;10:1470–1476.
[16] Couch Y, Buzàs EI, Di Vizio D, et al. A brief history of nearly EV-erything - the rise and rise of extracellular vesicles. J Extracell Vesicles 2021;10: e12144.
[17] Mathieu M, Martin-Jaular L, Lavieu G, et al. Specificities of secretion and uptake of exosomes and other extracellular vesicles for cell-to-cell communication. Nat Cell Biol 2019;21:9–17.
[18] **Yáñez-Mó M**, **Siljander PR**, Andreu Z, et al. Biological properties of extracellular vesicles and their physiological functions. J Extracell Vesicles 2015;4:27066.
[19] Quaglia M, Dellepiane S, Guglielmetti G, et al. Extracellular vesicles as mediators of cellular crosstalk between immune system and kidney graft. Front Immunol 2020;11:74.
[20] An Q, van Bel AJE, Hückelhoven R. Do plant cells secrete exosomes derived from multivesicular bodies? Plant Signaling Behav 2007;2:4–7.
[21] Johnstone RM, Mathew A, Mason AB, et al. Exosome formation during maturation of mammalian and avian reticulocytes: evidence that exosome release is a major route for externalization of obsolete membrane proteins. J Cell Physiol 1991;147:27–36.
[22] Skokos D, Botros HG, Demeure C, et al. Mast cell-derived exosomes induce phenotypic and functional maturation of dendritic cells and elicit specific immune responses in vivo. J Immunol 2003;170:3037–3045.
[23] van Niel G, D'Angelo G, Raposo G. Shedding light on the cell biology of extracellular vesicles. Nat Rev Mol Cell Biol 2018;19:213–228.
[24] Alqurashi H, Ortega Asencio I, Lambert DW. The emerging potential of extracellular vesicles in cell-free tissue engineering and regenerative medicine. Tissue Eng B Rev 2021;27:530–538.
[25] Taverna S, Pucci M, Alessandro R. Extracellular vesicles: small bricks for tissue repair/regeneration. Ann Transl Med 2017;5:83.
[26] Zhang X, Yuan X, Shi H, et al. Exosomes in cancer: small particle, big player. J Hematol Oncol 2015;8:83.
[27] **Julich-Haertel H**, **Urban SK**, **Krawczyk M**, et al. Cancer-associated circulating large extracellular vesicles in cholangiocarcinoma and hepatocellular carcinoma. J Hepatol 2017;67:282–292.
[28] Willms AG, Krawczyk M, Julich-Haertel H, et al. What we missed then, AI sees now: revisiting legacy large extracellular vesicle data to reveal synergistic biomarkers for liver cancer screening. JHEP Rep 2025;7:101540.
[29] **Urban SK**, **Sanger H**, **Krawczyk M**, et al. Synergistic effects of extracellular vesicle phenotyping and AFP in hepatobiliary cancer differentiation. Liver Int 2020;40:3103–3116.
[30] **Wang B**, **Wohler A**, Greven J, et al. Liquid biopsy in organ damage: small extracellular vesicle chip-based assessment of polytrauma. Front Immunol 2023;14:1279496.
[31] Wohler A, Gries SK, Salzmann RJS, et al. Monocyte derived large extracellular vesicles in polytrauma. J Extracell Biol 2024;3:e70005.
[32] Mayr C, Wagner A, Stoecklinger A, et al. 3-Deazaneplanocin A may directly target putative cancer stem cells in biliary tract cancer. Anticancer Res 2015;35:4697–4705.
[33] Thery C, Witwer KW, Aikawa E, et al. Minimal information for studies of extracellular vesicles 2018 (MISEV2018): a position statement of the International Society for Extracellular Vesicles and update of the MISEV2014 guidelines. J Extracell Vesicles 2018;7:1535750.
[34] Lotvall J, Hill AF, Hochberg F, et al. Minimal experimental requirements for definition of extracellular vesicles and their functions: a position statement from the International Society for Extracellular Vesicles. J Extracell Vesicles 2014;3:26913.
[35] Singal AG, Llovet JM, Yarchoan M, et al. AASLD Practice Guidance on prevention, diagnosis, and treatment of hepatocellular carcinoma. Hepatology 2023;78:1922–1965.
[36] **Sounderajah V**, **Guni A**, Liu X, et al. The STARD-AI reporting guideline for diagnostic accuracy studies using artificial intelligence. Nat Med 2025;31:3283–3289.
[37] Schima W, Heiken J. LI-RADS v2017 for liver nodules: how we read and report. Cancer Imaging 2018;18:14.
[38] Gu X, Yin G, Lee JJ. Bayesian two-step Lasso strategy for biomarker selection in personalized medicine development for time-to-event endpoints. Contemp Clin Trials 2013;36:642–650.
[39] Diaz-Uriarte R, Gomez de Lope E, Giugno R, et al. Ten quick tips for biomarker discovery and validation analyses using machine learning. Plos Comput Biol 2022;18:e1010357.
[40] Taguchi YH, Murakami Y. Principal component analysis based feature extraction approach to identify circulating microRNA biomarkers. PLoS ONE 2013;8:e66714.
[41] Jang ES, Jeong SH, Kim JW, et al. Diagnostic performance of alpha-fetoprotein, protein induced by vitamin K absence, osteopontin, Dickkopf-1 and its combinations for hepatocellular carcinoma. PLoS ONE 2016;11:e0151069.
[42] Tayob N, Kanwal F, Alsarraj A, et al. The performance of AFP, AFP-3, DCP as biomarkers for detection of hepatocellular carcinoma (HCC): a Phase 3 biomarker study in the United States. Clin Gastroenterol Hepatol 2023;21:415–423.
[43] Best J, Bilgi H, Heider D, et al. The GALAD scoring algorithm based on AFP, AFP-L3, and DCP significantly improves detection of BCLC early stage hepatocellular carcinoma. Z Gastroenterol 2016;54:1296–1305.
[44] Hudson D, Afzaal T, Bualbanat H, et al. Modernizing metabolic dysfunction-associated steatotic liver disease diagnostics: the progressive shift from liver biopsy to noninvasive techniques. Therap Adv Gastroenterol 2024;17:17562848241276334.
[45] **Berger D**, **Desai V**, Janardhan S. Con: liver biopsy remains the gold standard to evaluate fibrosis in patients with nonalcoholic fatty liver disease. Clin Liver Dis 2019;13:114–116.
[46] **Liang Y**, **Xu F**, **Guo Y**, et al. Diagnostic performance of LI-RADS for MRI and CT detection of HCC: a systematic review and diagnostic meta-analysis. Eur J Radiol 2021;134:109404.
[47] **Liu X**, **Ni X**, **Li Y**, et al. Diagnostic performance of LI-RADS version 2018 for primary liver cancer in patients with liver cirrhosis on enhanced MRI. Front Oncol 2022;12:934045.
[48] Guan MC, Zhang SY, Ding Q, et al. The performance of GALAD score for diagnosing hepatocellular carcinoma in patients with chronic liver diseases: a systematic review and meta-analysis. J Clin Med 2023;12:949.
[49] Istemihan Z, Senkal IV, Karaca D, et al. The role of GALAD score in the surveillance of hepatocellular carcinoma. Sci Rep 2025;15:22279.
[50] Nishida-Aoki N, Tominaga N, Takeshita F, et al. Disruption of circulating extracellular vesicles as a novel therapeutic strategy against cancer metastasis. Mol Ther 2017;25:181–191.
[51] Tejera E, Rocha-Perugini V, Lopez-Martin S, et al. CD81 regulates cell migration through its association with Rac GTPase. Mol Biol Cell 2013;24:261–273.
[52] Kumar MA, Baba SK, Sadida HQ, et al. Extracellular vesicles as tools and targets in therapy for diseases. Signal Transduction Targeted Ther 2024;9:27.
[53] Li D, Wang L, Jiang B, et al. Improving cancer immunotherapy by preventing cancer stem cell and immune cell linking in the tumor microenvironment. Biomed Pharmacother 2024;170:116043.

[54] **Sirica AE**, **Gores GJ**, **Groopman JD**, et al. Intrahepatic cholangiocarcinoma: continuing challenges and translational advances. Hepatology 2019;69:1803–1815.

[55] Lee YT, Wang JJ, Luu M, et al. Comparison of clinical features and outcomes between intrahepatic cholangiocarcinoma and hepatocellular carcinoma in the United States. Hepatology 2021;74:2622–2632.

Keywords: CD133; CD133/2; Cholangiocarcinoma; Hepatocellular carcinoma; Extracellular vesicles; Liquid biopsy; Liver cancer; Biomarker.

Received 23 October 2025; received in revised form 4 March 2026; accepted 6 March 2026; Available online 25 March 2026

Journal of Hepatology, Volume *8*

Supplemental information

AI-guided additive scoring model for differential diagnosis of primary liver cancer

Rebekka J.S. Salzmann, Tudor Mocan, Arnulf G. Willms, Angelina Klein, Robert Schwab, Emil Mois, Cristiana Grapa, Lavinia Patricia Mocan, Rares Craciun, Zeno Sparchez, Ingo G.H. Schmidt-Wolf, Jan Best, Hartmut H. Schmidt, Marcin Krawczyk, Simon C. Robson, Veronika Lukacs-Kornek, and Miroslaw T. Kornek

AI-guided additive scoring model for differential diagnosis of primary liver cancer

Rebekka J.S. Salzmann, Tudor Mocan, Arnulf G. Willms, Angelina Klein, Robert Schwab, Emil Mois, Cristiana Grapa, Lavinia Patricia Mocan, Rares Craciun, Zeno Sparchez, Ingo G.H. Schmidt-Wolf, Jan Best, Hartmut H. Schmidt, Marcin Krawczyk[7], Simon C. Robson, Veronika Lukacs-Kornek, Miroslaw T. Kornek

Table of contents

Supplementary figures……………………………………………………………………2

Supplementary tables……………………………………………………………….………3

Supplementary figures

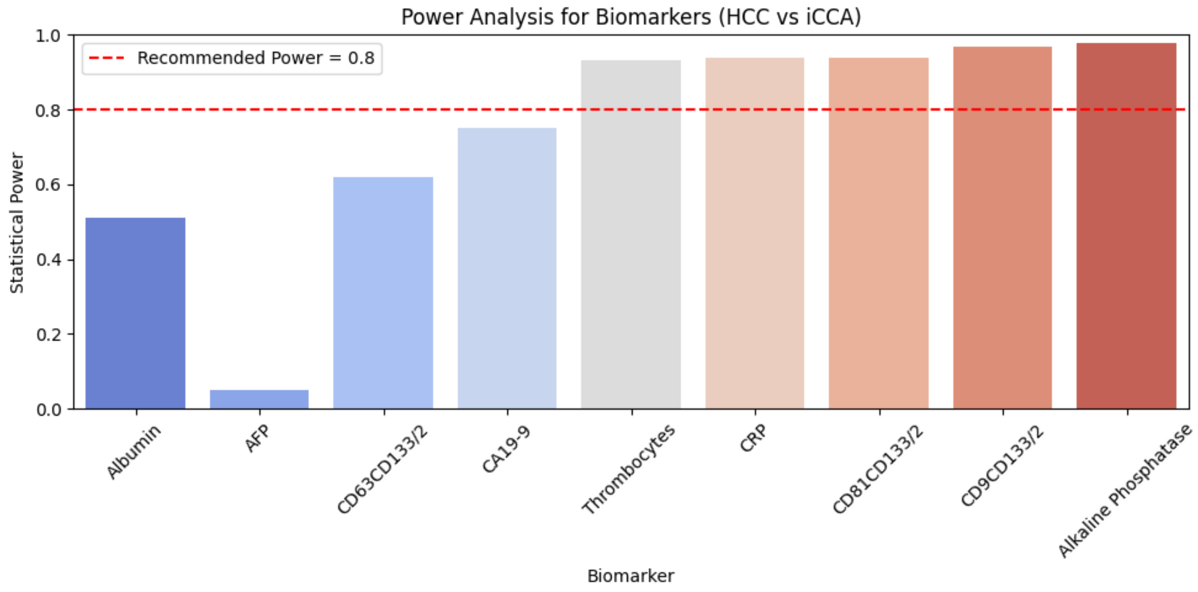

Fig. S1 Statistical power analysis for individual biomarkers (HCC vs iCCA). *The post-hoc power estimation was performed for each biomarker comparison using effect sizes derived from Mann–Whitney U tests (n = 25 per group). Bars indicate the achieved statistical power for detecting group differences between hepatocellular carcinoma (HCC) and intrahepatic cholangiocarcinoma (iCCA). The red dashed line marks the recommended threshold for adequate power (0.8). Most parameters—particularly Alkaline Phosphatase, $CD9^+CD133/2^+$, $CD81^+CD133/2^+$, CRP, and Thrombocytes—exceeded this threshold, indicating sufficient sensitivity to detect true differences. AFP and Albumin showed lower power due to high intra-group variability and limited effect size.*

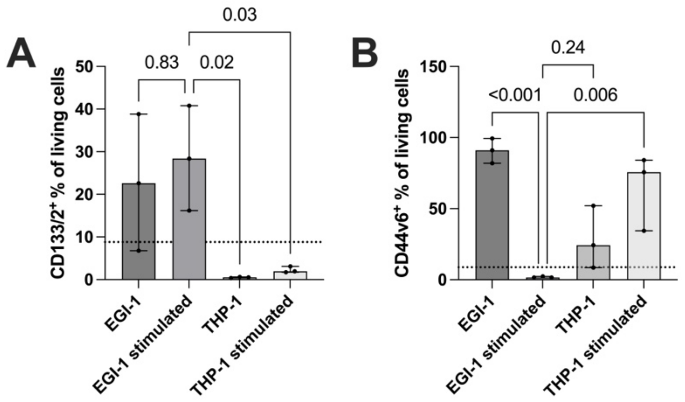

Fig. S2 Validation of CD133/2 and CD44v6 antibody candidates on EGI-1 cholangiocarcinoma cells and THP-1 monocytic cells. *Biological triplicates of unstimulated and stimulated EGI-1 cells and unstimulated and stimulated THP-1 cells were immunolabeled with candidate antibodies against CD133/2 (PE) and CD44v6 (APC) and analyzed by flow cytometry for surface expression. (A) CD133/2 surface expression on EGI-1 cells under standard culture conditions and after starvation (FBS deprivation), and on THP-1 cells under standard conditions and after cytokine stimulation. Starvation of EGI-1 cells resulted in a significant increase in CD133/2 expression compared to unstimulated EGI-1 cells ($p = 0.03$). No significant CD133/2 expression was detected on THP-1 cells under either condition ($p = 0.83$ and $p = 0.02$, respectively). (B) CD44v6 surface expression on EGI-1 and THP-1 cells. EGI-1 cells showed significantly higher CD44v6 expression compared to THP-1 cells under standard culture conditions ($p < 0.001$). Cytokine stimulation of THP-1 cells with IL-4 and IL-13 for 72 h resulted in a significant induction of CD44v6 expression compared to unstimulated THP-1 cells ($p = 0.006$), while no significant change was observed in EGI-1 cells following starvation ($p = 0.24$). Bars represent median values with 95% confidence intervals. Statistical analysis was performed using GraphPad Prism (version 10.4.1, GraphPad Software, USA). Group comparisons were conducted using one-way ANOVA assuming Gaussian distribution, followed by Dunnett's multiple comparisons post-test. All tests were two-sided with a confidence level of 95%.*

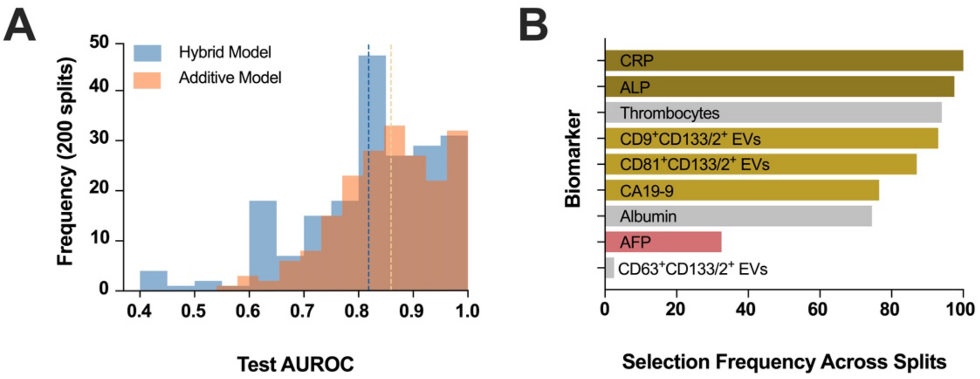

Fig. S3 Stability of diagnostic performance and feature selection across 200 repeated random train–test splits. *(A) Distribution of test AUROC values across 200 repeated random 80:20 train–test splits for the hybrid logistic regression model (continuous predictors) and the translated additive scoring model (binarized ROC–Youden thresholds). Dashed vertical lines indicate the mean test AUROC across splits for each model (hybrid mean = 0.818; additive mean = 0.859). (B) LASSO feature-selection frequency across the same 200 random splits, shown as the percentage of splits in which each biomarker was selected during model training. Core markers (CRP, alkaline phosphatase, thrombocytes, CD9$^+$CD133/2$^+$ EVs, CD81$^+$CD133/2$^+$ EVs) demonstrated consistently high selection frequencies, whereas AFP and CD63$^+$CD133/2$^+$ EVs were selected less frequently.*

Supplementary tables

Table S1 Patient demographics and baseline clinical characteristics of the iCCA and HCC cohorts. *Continuous variables are presented as median [interquartile range, IQR], and categorical variables as n (%). P-values for continuous variables were calculated using the two-sided Mann–Whitney U test; p-values for categorical variables were calculated using Fisher's exact test. Exact p-values are reported in the table. Blank cells indicate insufficient data for statistical comparison. Barcelona Clinic Liver Cancer (BCLC) classification applies only to HCC patients, while American Joint Committee on Cancer (AJCC) staging applies only to iCCA patients.*

Variable	iCCA (n=25)	HCC (n=25)	p-value
Age	64.00 [58.25–68.00]	66.00 [61.25–70.75]	0.42
CRP	2.83 [0.88–7.83]	0.59 [0.43–0.77]	0.001
ALP	690.00 [456.00–1377.00]	271.00 [212.00–380.00]	<0.001
Albumin	3.50 [3.20–3.80]	3.70 [3.41–4.00]	0.05
Thrombocytes	231.00 [187.00–279.00]	135.00 [90.00–178.00]	<0.001
AFP	3.90 [2.70–21.00]	16.70 [6.80–57.70]	0.93
CA19-9	150.80 [19.70–200.00]	11.40 [5.70–56.70]	0.005
Gender: male	17 (68.0%)	16 (64.0%)	1.00
Gender: female	8 (32.0%)	9 (36.0%)	1.00
BCLC A		52.0%	
BCLC B		B: 20.0%	
BCLC C		C: 16.0%	
BCLC D		D: 12.0%	
AJCC stages	IB: 12.0%; II: 8.0%; IIIA: 4.0%; IIIB: 16.0%; IV: 36.0%; IVA: 8.0%; IVB: 16.0%		

Table S2 List of antibodies and isotypes used for cell- surface flow cytometric analysis and SP-IRIS*. The antibodies were titrated against their matching isotype controls before use and applied according to the manufacturers' instructions.*

Antibody (anti-human)	Conjugate	Clone	Catalog#	Vendor	Concentration [µg/µL]
CD133/2	PE	REA820	130-112-195	Miltenyi Biotec	0.15
Isotype	PE	REA293	130-118-347	Miltenyi Biotec	0.15
CD44v6	APC	REA706	130-111-238	Miltenyi Biotec	0.1
Isotype	APC	REA293	130-113-446	Miltenyi Biotec	0.02

Table S3. Data completeness across biomarkers and cohorts: *Overview of data completeness for all biomarkers included in the analysis, stratified by disease cohort (HCC and iCCA). Expected indicates the theoretical maximum number of measurements per cohort (n = 25 each), while Available reflects the number of measurements present in the final analysis dataset. Missing values were limited and primarily affected routine serological parameters, whereas all extracellular vesicle–derived markers were fully available across both cohorts.*

Biomarker	iCCA Expected (n=25)	iCCA Available (n)	HCC Expected (n=25)	HCC Available (n)	Missing total (n)
AFP	25	25	25	23	2
Albumin	25	25	25	25	0
ALP	25	25	25	25	0
CA19-9	25	22	25	22	6
CRP	25	25	25	25	0
Thrombocytes	25	25	25	25	0
$CD9^{+}CD133/2^{+}$ EVs	25	25	25	25	0
$CD63^{+}CD133/2^{+}$ EVs	25	25	25	25	0
$CD81^{+}CD133/2^{+}$ EVs	25	25	25	25	0

Table S4 Supplementary Table 4. Diagnostic performance of hybrid logistic regression models combining extracellular vesicle–derived and routine serological biomarkers for differentiation of HCC and iCCA. *AUROC values were computed in Python (scikit-learn) from receiver operating characteristic (ROC) curves of the respective model scores. Sensitivity and specificity were derived from the confusion matrix at the ROC-derived Youden-index threshold. P-values were calculated using a two-sided Pearson chi-square test applied to the 2×2 contingency table of predicted class versus histopathological ground truth (TP/FN/FP/TN), testing for independence between model prediction and disease entity.*

Model	AUROC	Sensitivity	Specificity	Chi-square p-value
With AFP (LASSO/PCA)	0.857	0.714	1.000	0.050
Without AFP (LASSO/PCA)	0.905	0.857	1.000	0.050

Table S5 Performance metrics of the additive scoring system derived from the LASSO/PCA hybrid models for differentiation of iCCA from HCC in LR-M lesions. *AUROC values were computed in Python (scikit-learn) from ROC curves of the respective total additive scores. The optimal total score cutoff for each model was determined using Youden's index (maximum of TPR – FPR). Sensitivity and specificity were calculated from the resulting confusion matrix at the selected cutoff. P-values were obtained using a two-sided Pearson chi-square test applied to the 2×2 contingency table of predicted class versus histopathological ground truth (TP/FN/FP/TN), testing for independence between model-based classification and disease entity.*

Model	AUROC	Sensitivity	Specificity	Chi-square p-value
With AFP (LASSO/PCA Final Model)	0.962	0.76	1.000	<0.0001
Without AFP (LASSO/PCA Final Model)	0.952	0.76	1.000	<0.0001

Table S6 Monte Carlo robustness analysis of the PRISM-based 5-point additive scoring system. *To assess the stability of the PRISM-derived score under measurement variability, marker values were perturbed by multiplicative uniform noise at ±5%, ±10%, and ±20% (independently per marker and per sample). For each noise level, 500 Monte Carlo iterations were performed. In each iteration, the total additive score was recalculated using the fixed PRISM cut-offs, and AUROC was recomputed from*

the resulting total scores. The table reports mean AUROC, standard deviation, and minimum/maximum AUROC across iterations. No hypothesis testing was performed; therefore, no p-values apply.

Noise Level	Mean AUROC	SD (AUROC)	Min AUROC	Max AUROC
±5 %	0.894	0.008	0.877	0.914
±10 %	0.894	0.010	0.87	0.925
±20 %	0.891	0.015	0.846	0.933

Table S7. Bootstrap-derived uncertainty of cohort-derived ROC–Youden cut-offs (2,000 iterations). *Cut-off stability was evaluated using stratified bootstrap resampling (2,000 iterations). For each biomarker, the table reports the median bootstrap-derived cut-off, the 2.5% and 97.5% percentile interval, and the percentage of bootstrap iterations in which the direction of effect (higher values associated with iCCA) remained consistent. Stability classification reflects combined assessment of directional consistency and relative dispersion of the bootstrap cut-off distribution. AFP was included for completeness but is fixed at 20 ng/mL in guideline-based analyses.*

Biomarker	Bootstrap Median Cut-off	2.5% Percentile	97.5% Percentile	Direction Consistency (%)	Bootstrap Stability
AFP	6.7	2.8	32.0	7.7	unstable
Albumin	3.5	3.0	4.0	2.6	unstable
ALP	456.0	456.0	912.0	100.0	stable
CA19-9	117.9	3.6	171.1	95.0	unstable
Thrombocytes	167.0	166.0	217.0	100.0	stable
$CD9^{+}CD133/2^{+}$ EVs	5292000.0	2168633.3	11687000.0	100.0	stable
$CD81^{+}CD133/2^{+}$ EVs	1582000.0	161000.0	3700000.0	100.0	moderate

Table S8 Cox proportional hazards models and assessment of proportional hazards assumptions for CD133/2-positive small extracellular vesicle subpopulations in LR-M patients. *Univariable Cox proportional hazards models were performed separately within HCC and iCCA subgroups (n = 25 each) to evaluate the association between above- versus ≤-median small extracellular vesicle (EV) levels and overall survival. Hazard ratios (HR) with 95% confidence intervals (CI) are shown. The proportional hazards (PH) assumption was formally tested using Schoenfeld residual–based tests (rank transformation). Corresponding p-values are reported. A p-value > 0.05 indicates no evidence of violation of the PH assumption. No significant deviations from proportional hazards were detected in any model.*

Model	n	Events	HR	95% CI	Schoenfeld p	PH violated?
HCC $CD9^{+}CD133/2^{+}$	25	20	1.89	0.76–4.70	0.444	No
HCC $CD81^{+}CD133/2^{+}$	25	20	2.88	1.11–7.43	0.893	No
iCCA $CD9^{+}CD133/2^{+}$	25	23	3.89	1.43–10.58	0.586	No
iCCA $CD81^{+}CD133/2^{+}$	25	23	2.16	0.87–5.34	0.337	No
